# Obesity and psychology: a bibliometric analysis of half a century

**DOI:** 10.3389/fnut.2025.1539587

**Published:** 2025-04-16

**Authors:** Haiqiu Zhou, Shan Liu, Zixuan Xiao, Saiqiong Yin, Boyan Fan, Guixiang Sun

**Affiliations:** College of Traditional Chinese Medicine, Hunan University of Traditional Chinese Medicine, Changsha, China

**Keywords:** psychology, obesity, bibliometrics, CiteSpace, VOSviewer

## Abstract

**Background:**

The interaction mechanisms between obesity and psychological factors are intricate and bidirectional. Psychological issues can prompt unhealthy eating behaviors, impede weight management efforts, and elevate the risk of obesity. This study employs bibliometric approaches to conduct a comprehensive analysis of the knowledge structure, research hotspots, and development trends in the field of obesity and psychology, offering valuable references for future research in this area.

**Methods:**

This study draws on the Web of Science Core Collection (WoSCC) database, with “obesity” and “psychology” serving as the primary search terms. Leveraging CiteSpace (version 6.3.R1) and VOSviewer (version 1.6.20) software, bibliometric analyses were conducted on various indicators, including the number of publications, publication volume, authors, journals, references, countries, institutions, and keywords. Through co-citation analysis and keyword co-occurrence analysis, the research hotspots and developmental trajectories in this field were revealed.

**Results:**

Based on the inclusion and exclusion criteria, a total of 2,753 relevant articles were ultimately included in this study. The results indicate that since the 21st century, there has been a significant surge in the number of publications in the field of obesity and psychology. Developed countries like the United States, Canada, the United Kingdom, and Australia are at the forefront of this field. Leading research institutions include Yale University, University College London, and the University of Pennsylvania. Among the authors, GRILO CM has the highest publication output. Research hotspot keywords primarily include “depression,” “stress,” “emotional eating,” “bariatric surgery,” “intervention,” “weight stigma,” and “self-regulation.” Current research trends reveal a marked regional imbalance in international collaboration in the field of obesity and psychology. In particular, there exists a notable absence of substantive cooperation between developed and developing countries. Research hotspots mainly center around the following aspects: Firstly, it focuses on the prevalence of common psychological distress symptoms, including depression, anxiety, and stress, within the obese population and the implications these symptoms have for health. Secondly, mental health issues like binge eating and emotional eating play a pivotal role in the onset and maintenance of obesity. Thirdly, psychosocial factors like health-related quality of life and weight stigma are at the core of obesity intervention and have potential impacts on behavioral change. Meanwhile, researchers are increasingly concentrating on the individualized mental health requirements of obese populations, emphasizing the importance of evidence-based psychological interventions in the management of obesity. These research hotspots not only enhance our understanding of the complex relationship between obesity and mental health but also provide crucial theoretical foundations and practical insights for future research directions.

**Conclusion:**

This study employs bibliometric approaches to conduct a comprehensive and in-depth analysis of research trends and developments in the field of obesity and psychology. The research reveals the current status and characteristics of this field from multiple perspectives, offering scientific backing for researchers to identify potential collaborators, pinpoint hotspot issues, and keep abreast of the latest developments. Looking forward to the future, related research can further expand data sources, diversify research viewpoints, and delve more profoundly into the complex relationship between obesity and mental health.

## 1 Introduction

Over the past five decades, global obesity rates have almost tripled (1). In most developed countries, the average weight of the population has increased substantially. Obesity not only exerts adverse impacts on the crucial health systems of the human body, especially the cardiovascular and metabolic systems, leading to a reduced life expectancy, but it also poses associated risks to mental health (2, 3). The relationship between obesity and mental health is highly complex (4, 5). On one hand, obesity can lead to mental health problems such as stress, depression, anxiety, stigma, impulsivity, and low self-esteem. These psychological issues can, in turn, interfere with an individual’s eating behavior and weight management, thus creating a vicious cycle. On the other hand, mental health problems may also increase an individual’s risk of obesity. Studies have shown that depression and anxiety may cause individuals to overeat or have a greater inclination to choose high-calorie foods, resulting in weight gain (6–8). Additionally, psychological factors such as stress and emotional regulation ability may also affect an individual’s weight management (4).

Mood has an important impact on diet. Positive emotions like happiness and satisfaction may encourage people to select healthy foods and maintain a moderate dietary intake. Conversely, negative emotions such as stress, anxiety, and depression may drive individuals to alleviate their feelings by overeating or opting high-calorie, high-fat foods (9, 10). Moreover, emotions can also affect an individual’s perception and evaluation of food, making them more likely to prefer high-calorie, high-fat options. Therefore, effective emotional management is of great significance for an individual’s eating behavior and weight management (11, 12). Psychological interventions for obesity are challenging and often ineffective, as it involves complex personal, family, and social factors. Identifying the existence and significance of psychosocial and environmental risk factors seems to be an important element in preventing and treating obesity and its associated complications.

As far as we know, there is currently no bibliometric research that evaluates the research hotspots and trends in the field of obesity and psychology. This study aims to use bibliometric methods to systematically sort out research on obesity and psychology. The goal is to comprehensively understand, thoroughly compare, and meticulously analyze the research hotspots and development trajectories in this field. It is anticipated that this will offer a more comprehensive perspective for formulating obesity intervention strategies, promote mental health management among the obese population, and improve their overall health status.

## 2 Materials and methods

### 2.1 Data sources and methods

We selected the WoSCC (Science Citation Index Expanded, SCI-E) as the data source for our literature search. The specific search query formula was as follows: (ALL = “Obesity” OR ALL = “Overweight”) AND (ALL = “Psychology” OR ALL = “Psychological” OR ALL = “Psychological Factor” OR ALL = “Psychological Side Effect”). The time span for the search was set from the inception of the library up to October 20, 2024. To avoid the discrepancies caused by daily database updates, all searches were conducted on October 20, 2024. To guarantee the reliability of the research results, we assigned two independent reviewers to screen out studies that did not meet the inclusion criteria by reading the titles and abstracts. In case of a disagreement between the two reviewers, a third reviewer would make the final decision.

**Figure 1:**
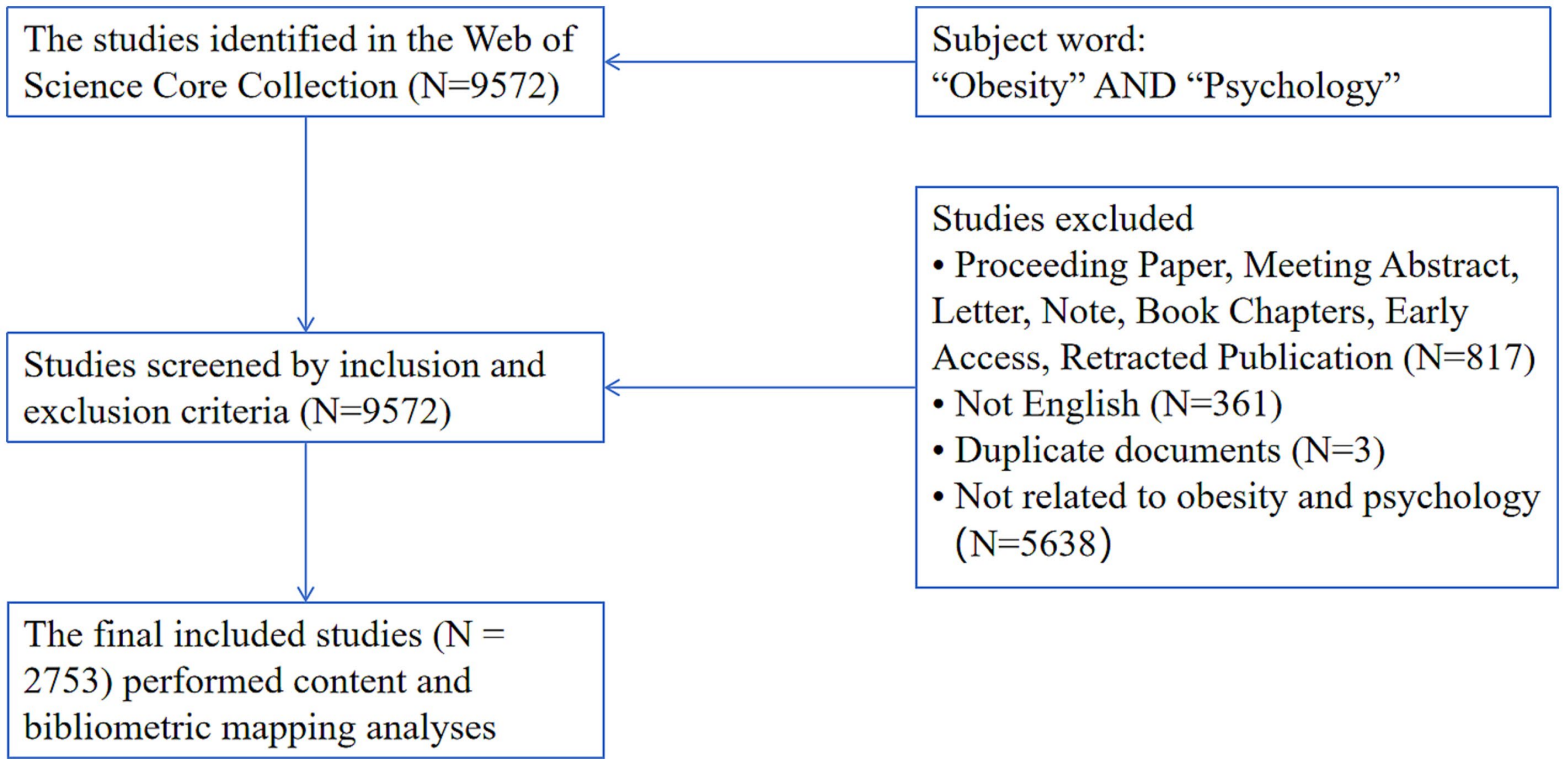
Screening flowchart.

Inclusion criteria: (1) Only articles and reviews were included. (2) The literature must be in English. Exclusion criteria: (1) Duplicate literature. (2) Literature that was not relevant to the research content of this study. (3) Literature in specific file types, such as meeting minutes, edited materials, letters, conference abstracts, book reviews, corrections, data papers, and retracted literature. (4) Non-English literature. After the search, a total of 9,572 records were retrieved, and 2,753 records were ultimately retained. Among these, there were 2,370 original articles and 383 review papers. The detailed screening process is shown in Figure 1.

### 2.2 Bibliometric methods

We employed Microsoft Excel 2019, CiteSpace (version 6.3.R1), VOSviewer (version 1.6.20), and R 4.4.1 for data presentation, analysis, and description. The measurement units involved included publications, authors, journals, references, countries, institutions, and keywords.

CiteSpace software is an information visualization tool developed by Professor Chaomei Chen. It was built upon citation analysis theory and programmed in the Java language. This software is not only capable of exploring the citation space but also performing co-occurrence analysis on knowledge units such as authors, institutions, and countries (13). VOSviewer is a free JAVA-based software developed by Leiden University in the Netherlands. It utilizes text mining technology to construct a co-occurrence network of important terms extracted from scientific literature databases. With VOSviewer, users can generate maps from network data and visualize and examine these maps (14). Each of these two software programs has its own distinct advantages and unique features. In the field of visual analysis, they complement one another effectively (15).

When evaluating scientific achievements, we employed the H-index as an objective indicator. The H-index represents a scenario where a researcher has a maximum of h articles that have been cited at least h times. Generally speaking, the higher a researcher’s H-index, the greater the influence of their papers (16). However, some studies have suggested that the H-index has certain limitations. It fails to fully take into account articles with extremely high citation rates, as well as those that have not been cited or have only a small number of citations. To overcome this problem, we introduced the G-index as an additional evaluation metric. The G-index can more objectively measure an author’s contribution, and this method was also utilized in our study (17, 18).

The impact factor (IF) is a scientific-to-metric index that aids in ranking, evaluating, classifying, and comparing journals (18). The data for the IF was provided by the Journal Citation Reports published in 2024. These metrics are key indicators for evaluating the contribution of articles.

## 3 Results

### 3.1 Search results

Based on the literature retrieval and screening process illustrated in Figure 1, this study systematically conducted a search of the WoSCC. As a result, a total of 2,753 research papers centered around obesity and mental health were comprehensively gathered. These papers were published from the inception of the database up to October 20, 2024. These articles covered 89 countries/regions. They were published in 655 academic journals and involved 3,184 research institutions as well as 11,349 authors. The influence of these papers is substantial, with a cumulative citation count reaching 113,948 and an average citation count of 41.39 per paper. This clearly indicates that the research achievements in this field carry significant academic weight.

### 3.2 Annual trends in the number of publications

**Figure 2:**
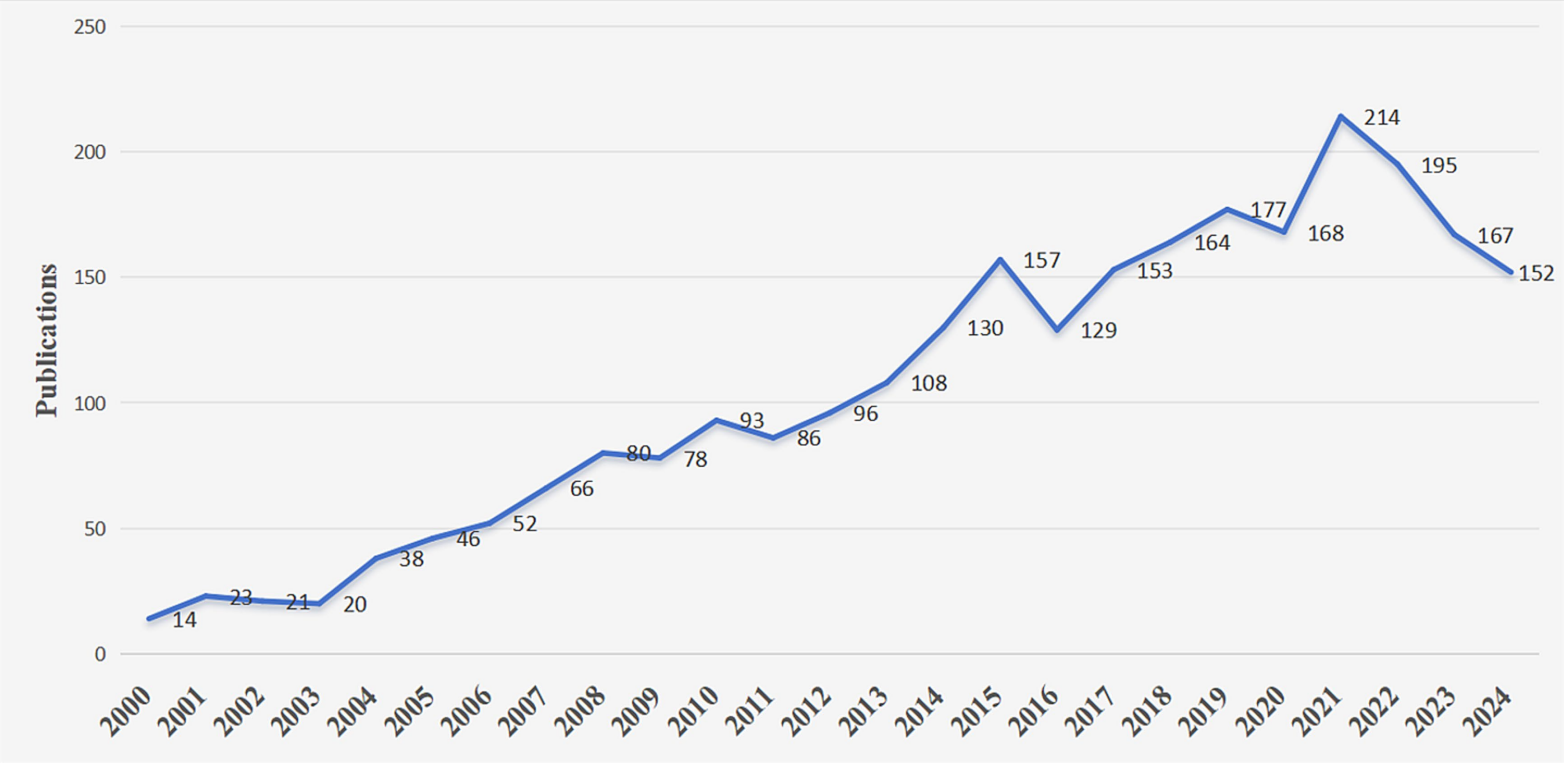
Publication growth trend of psychology and obesity from 2000 to 2024.

The annual number of publications serves as a critical metric for assessing the development pace and academic attention within a research domain. Literature analysis reveals that the first research paper in this field was published in 1975. However, prior to the 21st century, the annual number of published papers was relatively low, mirroring the limited research activities in this area during that period. In the 21st century, as the global obesity problem has become increasingly severe and there has been a growing focus on the relationship between obesity and mental health, the number of related studies has witnessed a significant surge. Figure 2 illustrates the annual publication trend in this field since 2000. Overall, the number of publications has been on a continuous upward trajectory, peaking in 2021 when a total of 214 papers were published. Although the number of publications has experienced a slight decline in recent years, it still remains at a high level, demonstrating sustained research momentum. This trend underscores the growing importance of obesity and mental health issues in both academia and society.

### 3.3 Country collaborative network

An analysis of the national publication collaboration network reveals the cooperation patterns and influence of various countries in the realm of obesity and mental health research. Table 1 lists the top 10 countries ranked by output. The United States takes the lead with 1,016 articles, accounting for 36.91%. It is followed by the United Kingdom (313, 11.37%), Australia (247, 8.97%), and Italy (230, 8.35%). In terms of total citations, the United States also ranks first (55,856, 49.03%), closely trailed by the United Kingdom (15,925, 13.97%) and Australia (12,611, 11.07%). Regarding the average citation count, the Netherlands has the highest figure (62.55), succeeded by the United States (54.97), Australia (51.05), and the United Kingdom (50.87). The low average citation frequency in Italy and China suggests that the quality of their publications requires improvement.

**Table 1:** Top 10 countries publications.

Rank	Country	Np	Tc	Average citation	h_Index	g_Index
1	United States	1,016	55,856	54.97	182	295
2	United Kingdom	313	15,925	50.87	92	161
3	Australia	247	12,611	51.05	93	151
4	Italy	230	6,872	29.88	58	95
5	Canada	158	7,238	45.81	60	118
6	Germany	151	7,028	46.5	83	122
7	Netherlands	126	7,882	62.55	63	124
8	China	115	2,023	17.59	60	118
9	Spain	106	3,367	31.76	51	82
10	France	81	2,588	31.95	42	72

**Figure 3:**
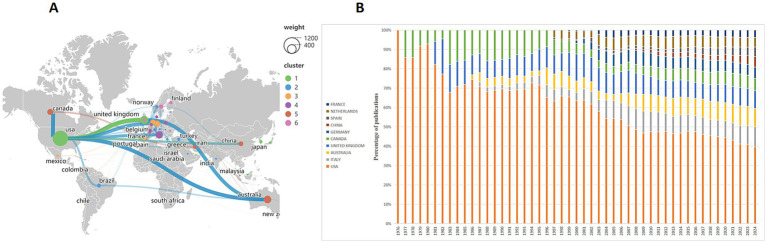
Analysis of countries publications. **(A)** Network diagram of collaborative relations between countries. Node sizes correspond to the number of publications, with distinct clusters indicated by different colors. The degree of cooperation is shown by the thickness of the connecting lines. **(B)** Percentage stacked area chart presents annual publications from the top 10 countries.

Using VOSviewer software, we generated a geographic collaboration network map (Figure 3A) with default thresholds. In the map, the size of each circle represents the number of publications, the lines indicate collaborative relationships, and the thickness of the lines reflects the strength of the collaboration. The VOS clustering method divided the network into six clusters. The map reveals that the United States collaborates most frequently with the United Kingdom and Australia, and also has varying levels of collaboration with Canada, Germany, and France. There is also close collaboration between the United Kingdom and Australia.

A percentage stacked area chart illustrates the relative trends of annual publications among the top 10 countries (Figure 3B). The United States, Canada, and the United Kingdom had relevant publications at an earlier stage, while Australia and Italy started publishing papers in 1987. In 1997, developed countries such as Spain, France, and the Netherlands also released research results. It’s worth noting that China has had publications in this field since 2003, but research in low-income countries remains relatively scarce. These findings suggest that developed countries paid early attention to this issue because of their material and economic bases. With economic development and the worsening of the obesity problem, research interest in this field is gradually growing and developing rapidly.

### 3.4 Organizations and journal analysis

Over 3,000 research institutions across the globe have made significant contributions to the realm of obesity and mental health. To highlight the most influential institutions, we have compiled a list of the top 10 based on publication volume (Table 2). Yale University in the United States takes the lead with 69 published articles, closely trailed by University College London in the United Kingdom with 52 articles. In terms of academic impact, gaged by the average citation rate, Yale University stands far ahead, boasting an average of 139.57 citations per article. The University of Minnesota in the United States ranks second, with an average of 101.05 citations per article.

**Table 2:** Top 10 organizations publications.

Rank	Organizations	Country	Np	Tc	Average citation
1	Yale Univ	United States	69	9,630	139.57
2	Ucl	UK	52	2,486	47.81
3	Univ Penn	United States	41	3,189	77.78
4	Monash Univ	Australia	40	2,921	73.03
5	Columbia Univ	United States	39	1,753	44.95
6	Univ Minnesota	United States	39	3,941	101.05
7	Kings Coll London	UK	37	1,199	32.41
8	Univ Toronto	Canada	36	1,576	43.78
9	Univ Leipzig	Germany	35	1,560	44.57
10	Deakin Univ	Australia	34	1,839	54.09

**Figure 4:**
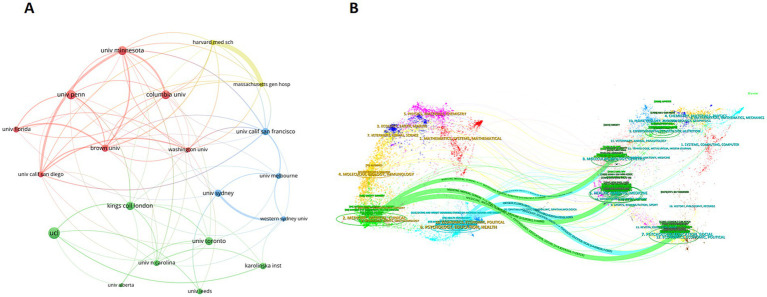
**(A)** The co-occurrence network of the top 20 corresponding organizations. The size of each node corresponds to the number of publications, while different colors denote distinct clusters. The line thickness demonstrates the extent of cooperation. **(B)** Dual map overlay of journals. The left side represents the fields of citing journals collection in the study, the right side represents the fields of cited journals collection, and the colored paths indicate the citation relationships.

When delving into the inter-agency partnerships, we constructed a network diagram of institutional collaboration co-occurrence (Figure 4A), which depicts the collaboration patterns of the 20 institutions that have made the most significant contributions. The diagram reveals that Harvard University has a close cooperation with Massachusetts General Hospital but has relatively less cooperation with other institutions. Seven universities, including the University of Minnesota, the University of Pennsylvania, Columbia University, the University of California, Brown University, the University of Florida, and the University of Washington, have formed the largest collaborative cluster, demonstrating a broad and close network of collaboration. This suggests that while some top institutions lead in research and impact, cross-institutional collaboration remains a crucial driving force for the development of this field.

A systematic analysis of journals in the field of obesity and psychology indicated that the journals making the greatest contributions were primarily concentrated in areas such as obesity, nutrition, and psychology, and some interdisciplinary journals. Table 3 lists the top 10 journals by publication volume. *Obesity Surgery* leads with 185 articles, followed closely by *Eating and Weight Disorders-Studies on Anorexia, Bulimia, and Obesity* (121 articles) and *International Journal of Obesity* (94 articles). Among the top 10 journals, only *Nutrients* and *Obesity Reviews* is an open-access (OA) journal. To facilitate academic exchange and knowledge sharing, researchers should be encouraged to publish their findings in OA journals to enhance the accessibility and impact of their research.

**Table 3:** Top 10 journals publications.

Rank	Journals	NP	IF	Partition	TC	h_Index	g_Index	OA
1	Obesity Surgery	185	2.9	q1	6,081	44	66	No
2	Eat Weight Disord-St	121	2.9	q1	2,017	24	37	No
3	Int J Obesity	94	4.2	q1	7,845	45	88	No
4	Appetite	88	4.7	q1	3,824	32	60	No
5	Health Psychology	78	3.1	q1	3,790	33	61	No
6	Nutrients	75	4.8	q1	1,067	19	30	Yes
7	Obesity	68	4.2	q1	5,064	36	68	No
8	Surg Obes Relat Dis	68	3.5	q1	1,685	23	38	No
9	Psychological Medicine	57	5.9	q1	2,585	29	50	No
10	Obesity Reviews	49	8.0	q1	3,820	31	49	Yes

Based on the total citation count of journals, *International Journal of Obesity* holds the top position with 7,845 citations. It has an H-index of 45 and a G-index of 88, which clearly indicates its substantial influence. *Obesity Surgery* (6,081 citations, H-index 44, G-index 66) and *Obesity* (5,064 citations, H-index 36, G-index 68) follow closely, suggesting that these three journals garner high attention and possess substantial academic influence in this field.

To explore interdisciplinary connections, we conducted a dual-map overlay analysis on journals. This analysis visually presents interdisciplinary interactions by overlaying two layers. Figure 4B displays a dual-map of journals in the field of obesity and psychology, where nodes represent journals. The map is divided into two parts. The left-hand side depicts the citing journals, whereas the right-hand side showcases the cited journals. The colored curves represent the citation paths. To quantify the differences in interdisciplinary research hotspots, we employed Z-score statistical calculations. The analysis reveals that research in medicine, psychiatry (green lines, *z* = 4.8254304, *f* = 28,315; *z* = 5.692812, *f* = 33,090; *z* = 2.9317217, *f* = 17,890), psychology, education, and health (blue lines, *z* = 4.0298004, *f* = 23,935; *z* = 3.2105556, *f* = 19,425) is significantly influenced by multiple disciplines. These disciplines encompass genetics, anatomy, nursing, social psychology, and educational psychology. This highlights the interdisciplinary nature of research in this field, as well as the robust knowledge flow and research interactions across different fields.

### 3.5 Author analysis

**Figure 5:**
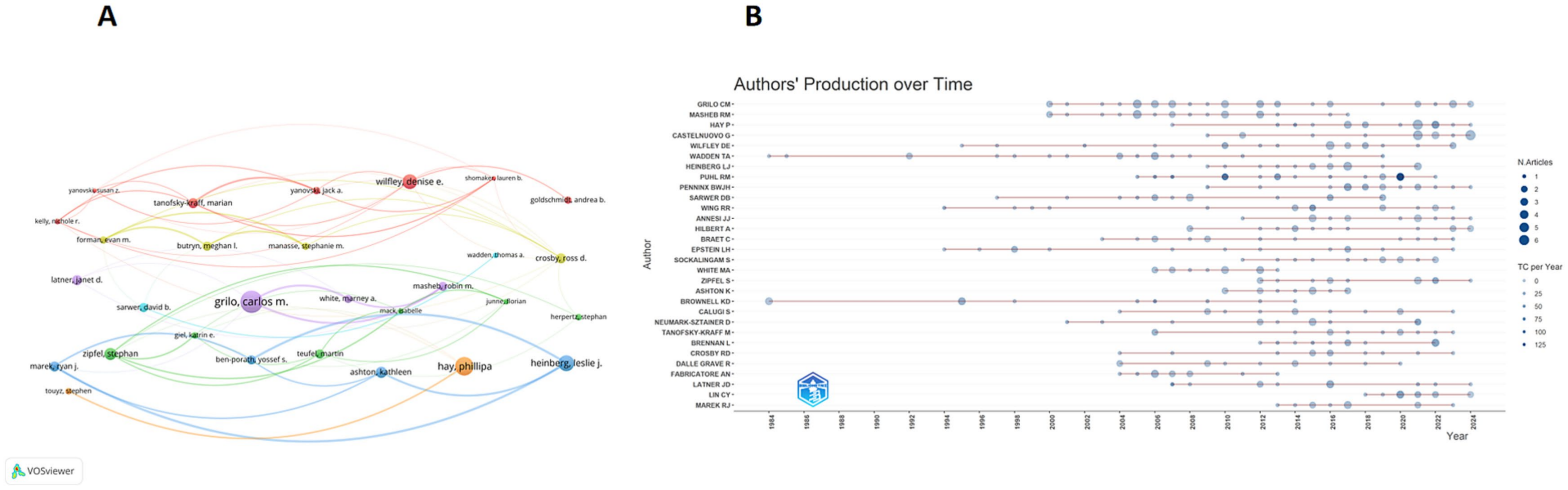
**(A)** The co-occurrence network of the top 30 authors of annual scientific publications. **(B)** The top 30 most productive authors over time, with the circle sizes reflecting the volume of their publications, and the color gradient signifying the annual number of citations.

We conducted an analysis of the author collaboration network and generated a co-occurrence map featuring the 30 most frequently collaborating authors (Figure 5A). Additionally, we illustrated the temporal trend of scientific productivity for the top 30 authors (Figure 5B). Temporally, the research contributions of the majority of authors were concentrated after 2004. The year 2020 stands out as an important milestone in this field, as the most influential articles were published during that year. Table 4 lists the top 10 authors in the H-index ranking. GRILO CM takes the first place with 37 related publications, achieving an H-index of 22 and a G-index of 37. MASHEB RM ranks second with 23 publications, achieving an H-index of 19 and a G-index of 23. Notably, both authors are from Yale University and have a close collaborative relationship. An analysis of their research findings reveals that their research interests primarily focus on postoperative management of weight-loss surgery (19, 20), eating disorders (21, 22), cognitive-behavioral therapy (23), and the application of weight-loss medications (24).

**Table 4:** Top 10 authors publications.

Rank	Authors	Np	Tc	Average Citation	h_Index	g_Index
1	Grilo CM	37	1,694	45.78	22	37
2	Masheb RM	23	1,301	56.56	19	23
3	Wadden TA	19	2,116		17	19
4	Puhl RM	17	4,509	265.23	16	17
5	Sarwer DB	15	1,476	98.4	14	15
6	Wilfley DE	20	1,293	64.65	14	20
7	Hay P	21	1,128	53.71	13	21
8	White MA	13	692	53.23	13	13
9	Heinberg LJ	17	423	24.88	12	17
10	Braet C	13	542	41.69	11	13

When measured by the total number of citations, PUHL RM ranks first with 4,509 citations, achieving an H-index of 16 and a G-index of 17. A highly cited article by PUHL RM (25) systematically reviewed existing research on weight stigma in children and adolescents. It focuses on the nature, extent, and primary sources of weight bias in obese adolescents, such as discriminatory behavior from peers, educators, and parents. The author also proposed future research directions, emphasizing the need to explore the impact of weight stigma on the physical health of adolescents and to develop intervention measures to improve societal attitudes. This article lays a theoretical foundation for the field and offers a scientific basis for policy-making and practical interventions.

### 3.6 Citation analysis

#### 3.6.1 References co-citation analysis

**Figure 6:**
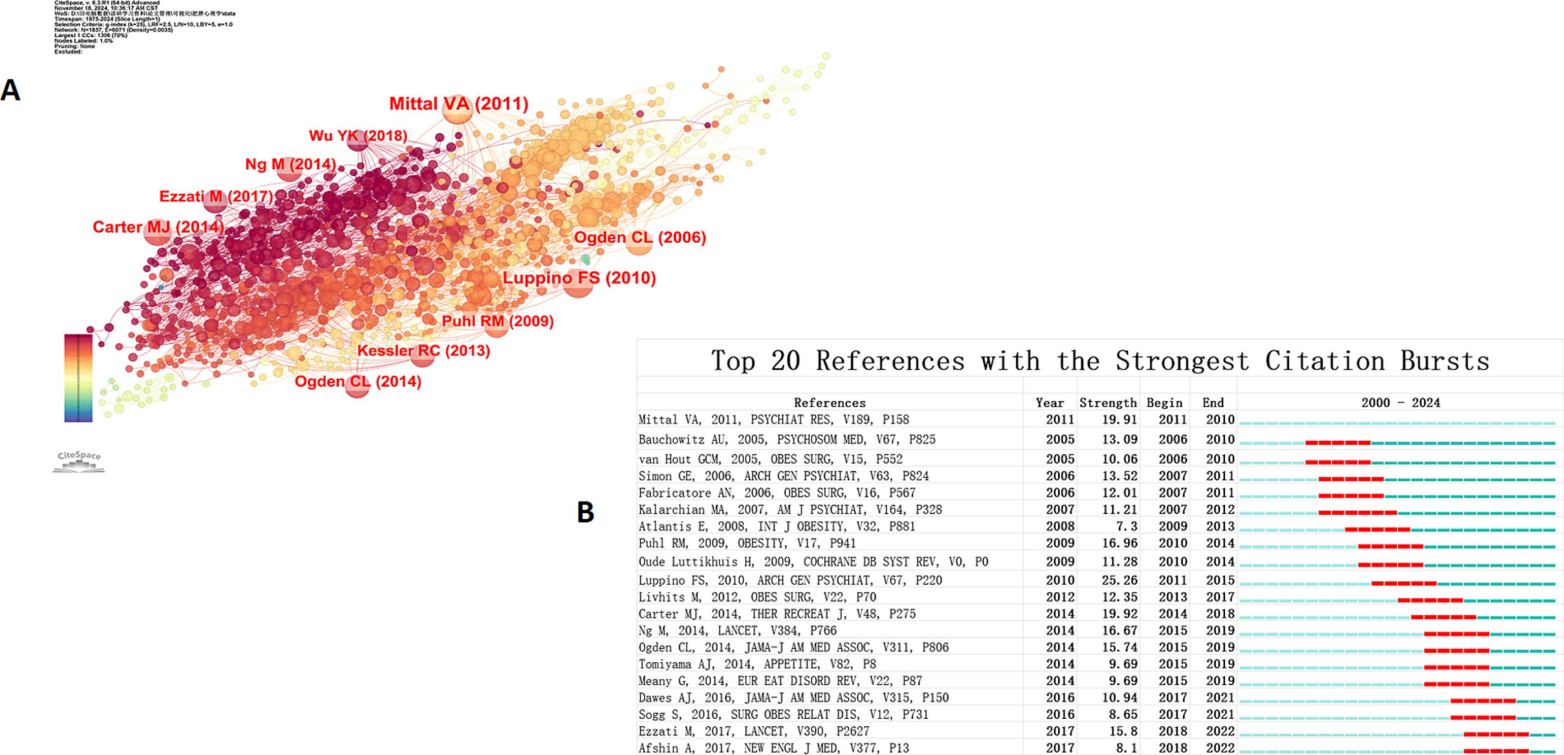
**(A)** Co-citation analysis of references. The node size corresponds to the number of citations, the link represents the co-cited relationship, and the left-to-right position and color of the node reflect the year of the cited article. **(B)** Burst detection analysis for the top 20 references of 2000 to 2024.

Co-citation refers to the phenomenon where two or more articles are cited simultaneously by subsequent papers. This phenomenon can reflect the knowledge structure and research frontiers of a field and uncover important research topics (26). Conducting a cluster analysis of co-cited literature can more clearly identify the hot topics and key issues in the research field. In this study, co-citation analysis was carried out using CiteSpace software. According to the default settings, 1,857 high-impact citations were extracted from the dataset. To enhance the precision of the analysis, documents with citation counts less than 1 were manually excluded. The final results are shown in Figure 6A. The labels in the figure display the top 10 most cited publications, their first authors, and the publication years. Meanwhile, Table 5 provides detailed information about these 10 highly cited documents.

**Table 5:** Top 10 cited references.

Rank	Authors	Title	Journals	Year	TC
1	Slavich GM	From stress to inflammation and major depressive disorder: a social signal transduction theory of depression	Psychol Bull	2014	1,310
2	Puhl RM	Obesity stigma: important considerations for public health	Am J Public Health	2010	1,180
3	Adam TC	Stress, eating and the reward system	Physiol Behav	2007	1,137
4	Puhl RM	Stigma, obesity, and the health of the nation's children	Psychol Bull	2007	884
5	Parsons TJ	Childhood predictors of adult obesity: a systematic review	Int J Obesity	1999	836
6	Phelan SM	Impact of weight bias and stigma on quality of care and outcomes for patients with obesity	Obes Rev	2015	756
7	Epel E	Stress may add bite to appetite in women: a laboratory study of stress-induced cortisol and eating behavior	Psychoneuroendocrino	2001	725
8	Stangl Al	The Health Stigma and Discrimination Framework: a global, crosscutting framework to inform research, intervention development, and policy on health-related stigmas	BMC Med	2019	713
9	Puhl RM	Confronting and coping with weight stigma: an investigation of overweight and obese adults	Obesity	2006	654
10	Greeno CG	Stress-induced eating	Psychol Bull	1994	615

The analysis reveals that among the top 10 most cited documents, 5 focused on weight stigma or weight stigmatization (25, 27–30), and 4 explored mechanisms related to depression (31) and stress-induced diet (32–34). The most cited document is the review titled “From stress to inflammation and major depressive disorder: a social signal transduction theory of depression,” which was published in *Psychological Bulletin* in 2014 (31). This review puts forward the theory of social signal transduction, which reveals the close association between the components of the immune system that mediate inflammation and major depressive disorder. It suggests that these mechanisms may share a common biological basis with inflammation-related diseases such as rheumatoid arthritis, chronic pain, obesity, diabetes, and cardiovascular disease. The author constructed a multi-level theoretical model and elaborated on the specific pathways through which social environmental stress triggers depression via neural, physiological, molecular, and genomic mechanisms.

The second most cited document is a review titled “Obesity stigma: important considerations for public health,” which was published in *Obesity Reviews* in 2010 (27). This document systematically analyzes the negative impact of weight stigmatization on the physical and mental health of obese individuals. It highlights that weight discrimination exacerbates health disparities and impedes the implementation of effective obesity interventions. It points out that weight stigmatization is not only a social justice issue but also a priority in the field of public health.

The third most cited document is a study titled “Stress, eating, and the reward system,” which was published in *Physiology and Behavior* in 2007 (29). This study proposes a reward-based stress - induced diet theory model. It emphasizes the critical roles of cortisol and the brain reward system in promoting high-calorie food consumption and explores the neuroendocrine relationship between stress and eating behavior. The study found that under stress, the brain reward pathway reduces the activity of the hypothalamic–pituitary–adrenal (HPA) axis by releasing opioids, thereby alleviating stress responses. However, under chronic stress, the balance of glucocorticoids, insulin, and leptin is disrupted, which may lead to overeating and visceral fat accumulation.

#### 3.6.2 Bursts of references

We employed the burst detection method to analyze the temporal distribution of references, with the aim of pinpointing the frequently-cited references within a specific period. This approach holds significant reference value in uncovering hot topics and research trends in the field. Figure 6B displays the top 20 documents with the highest burst strength, arranged chronologically. In the figure, the blue line represents the time span, and the red line represents the duration of burst citation. The analysis results indicate that the document with the strongest burst was published in 2010, boasting a burst strength of 25.26. This study (34) systematically explored the bidirectional relationship between depression and obesity, offering crucial insights into comprehending their interaction. The study discovered that obese individuals face a significantly elevated risk of depression, and this association is particularly pronounced among the American population and clinically diagnosed patients with depression. Additionally, the research indicated that depression itself serves as a significant predictor of obesity, suggesting that the two may interact via complex psychological, physiological, and social mechanisms. This finding not only deepens our understanding of the comorbidity of depression and obesity but also provides theoretical support for the development of related intervention strategies.

#### 3.6.3 Reference co-citation clustering

**Figure 7:**
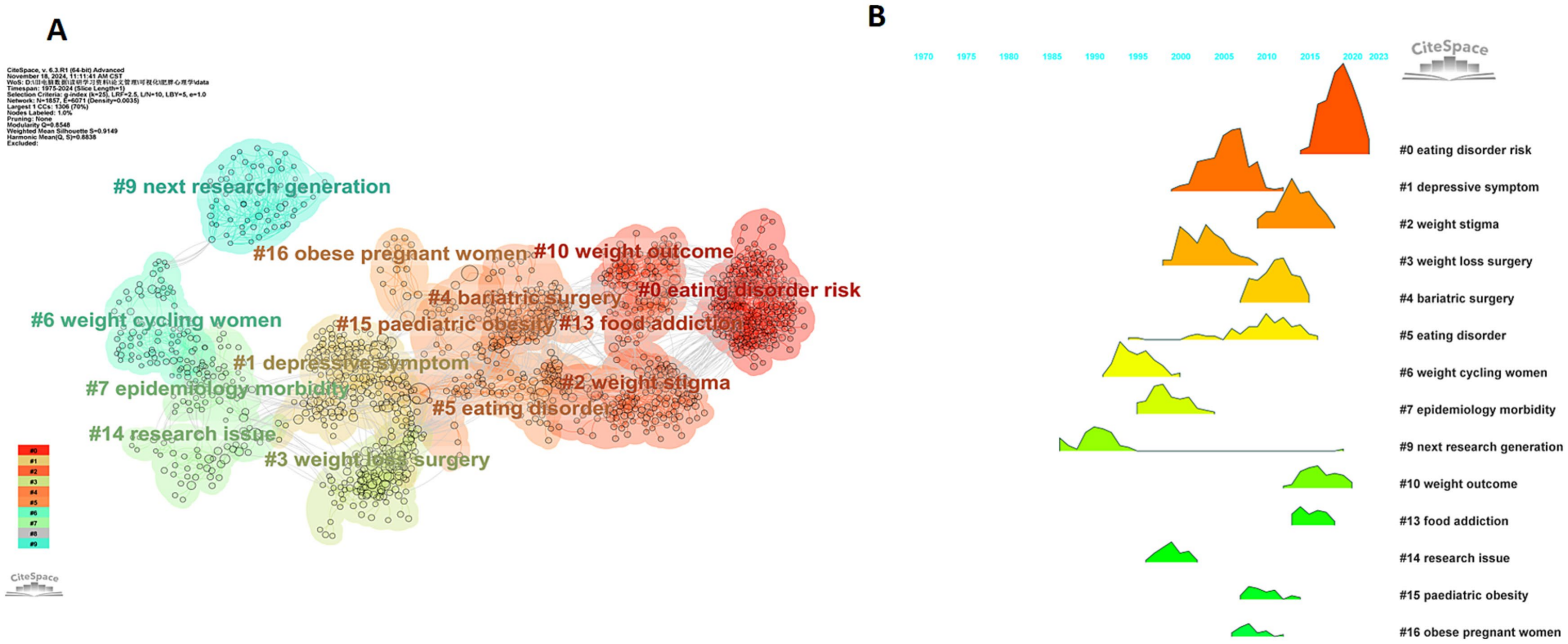
**(A)** The co-citation clustering network of references. **(B)** Ridgeline plot illustrating the co-citation clustering of references.

This study utilized the log-likelihood ratio (LLR) algorithm to extract keyword information from the titles of cited articles and constructed a co-citation clustering network (Figure 7A). The results revealed that the clustering module value Q was 0.8548 (Q > 0.3 indicated a significant clustering structure), and the average silhouette value S was 0.9149 (S > 0.5 indicated favorable clustering results), indicating that the clustering analysis yielded an ideal outcome. After a thorough examination of the 17 clusters extracted, we identified 14 clusters with well-defined research topics, including: #0 eating disorder risk, #1 depressive symptom, #2 weight stigma, #3 weight loss surgery, #4 bariatric surgery, #5 eating disorder, #6 weight cycling women, #7 epidemiology morbidity, #9 next research generation, #10 weight outcome, #13 food addiction, #14 research issue, #15 pediatric obesity, and #16 obese pregnant women. It is worth noting that clusters #8, #11, and #12 failed to form significant cluster links. This might be because of their low relevance within the network or because they were overlooked in the data. This study did not conduct further in-depth exploration of these clusters.

Figure 7B illustrates a visual map of the evolution of different research topics over time. The height and width of the peaks reflect the significance of each topic in different years. Each topic is assigned a specific color and accompanied by corresponding year annotations. Analysis has shown that prior to the 21st century, research primarily centered on topics such as epidemiological evidence of obesity and pregnant women. Since 2000, the focus has gradually shifted toward surgical treatments for severe obesity (such as bariatric surgery) and the association between depression and obesity. Around 2010, research concentrated more on the weight stigma experienced by obese patients. Around 2015, risk factors for eating disorders, such as overeating and food addiction, emerged as new hot topics. This trend suggests that psychological factors are playing an increasingly crucial role in the occurrence, development, and intervention of obesity. Literature co-citation analysis not only reveals the dynamic evolution of the main research topics but also promptly captures the cutting-edge developments in the research field. It provides a valuable theoretical foundation and practical guidance for formulating future research directions.

### 3.7 Keyword analysis

**Figure 8:**
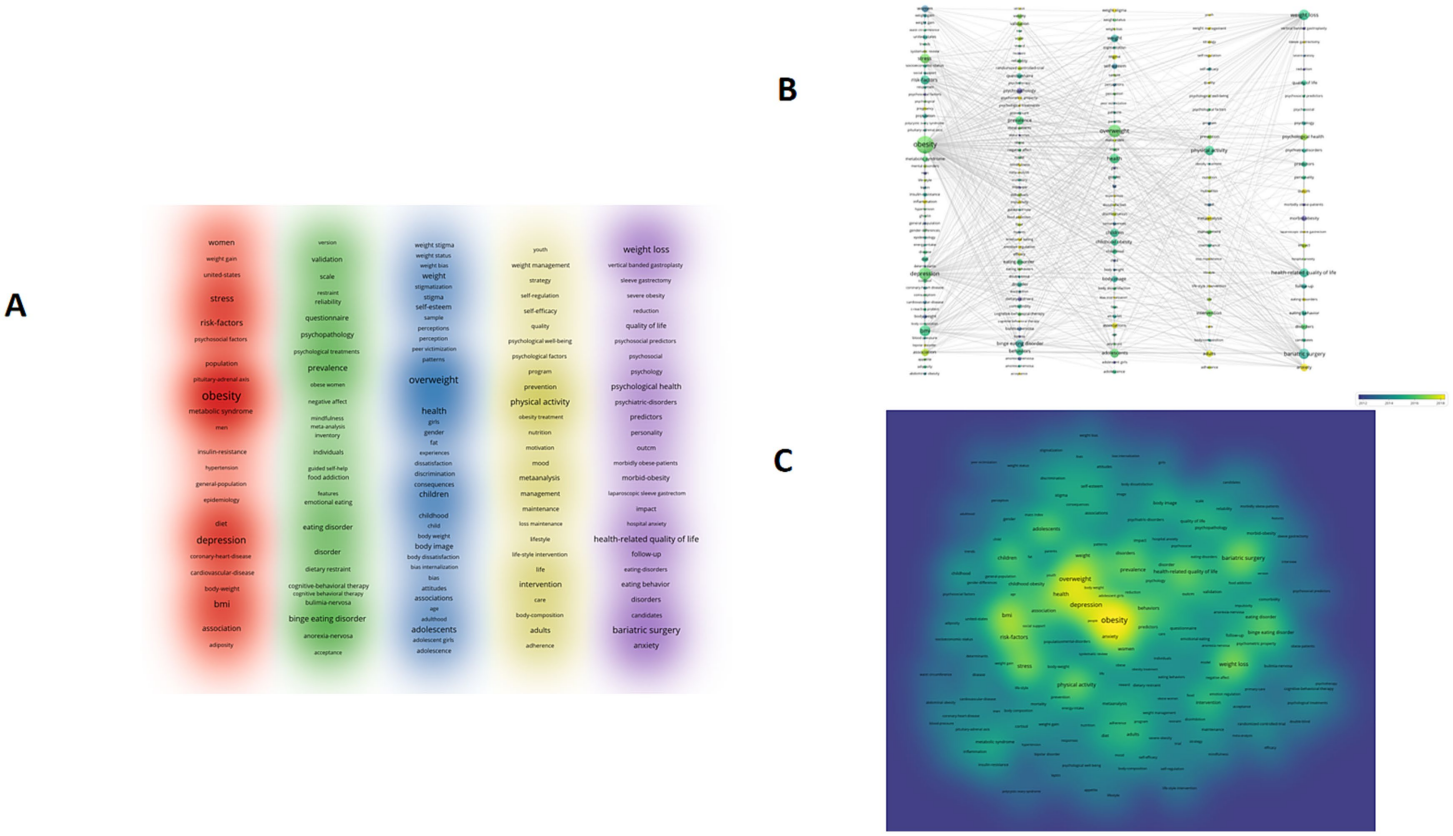
Keywords co-occurrence network analysis. **(A)** Keywords clustering visualization, with different colors representing distinct clusters. **(B)** Keywords cooccurrence and clustering overlay map, node sizes correspond to the frequency of the keywords, links symbolize co-occurrence relationships, and color signifies the average year of publications. **(C)** Keywords density visualization map, color indicates the concentration density of the study.

Keywords serve as crucial indicators for academic publication retrieval offering an intuitive reflection of research topics and substantially unveiling the main content and development trajectory of publications. Analyzing keyword frequency and trends can effectively help researchers grasp the hotspots and future directions within a research field. In this study the VOSviewer software system was employed to conduct an analysis of keywords associated with obesity and mental health research. Keywords with similar or identical meanings were grouped together to ensure the accuracy of the analysis. A minimum frequency threshold of 30 was set for keywords while default settings were used for other parameters to construct a keyword co-occurrence clustering network (Figure 8A). Additionally to reveal the temporal evolution characteristics of different research topics the color-coding of keywords was visualized based on the average publication year (Figure 8B). The distribution density and research focus of keywords were visually displayed through a density visualization network map (Figure 8C). From the perspective of time high-frequency keywords in earlier research such as “psychosocial factors,” “fat,” and “morbid-obesity,” have gradually given way to emerging themes like “lifestyle,” “emotional eating,” “inflammation,” “intervention,” “weight stigma,” and “self-regulation.” Based on the keyword frequency (Table 6) it is evident that “depression,” “stress,” and “anxiety” are the most common symptoms of psychological distress. Moreover “adolescents,” “children,” and “women” are the primary groups of concern in the context of obesity and psychological distress. As a high-frequency keyword, “bariatric surgery” implies that surgical therapy may be an important approach to alleviating the psychological distress of severely obese patients. Furthermore the frequent appearance of keywords such as “physical activity,” “health-related quality of life,” “risk-factors,” “binge eating disorder,” and “prevalence” further emphasizes the key areas of concern in the field of obesity and psychology especially the relationship between physical activity and mental health. Improving health-related quality of life is the primary objective of obesity intervention and binge eating disorder represents an important research direction for obesity-related psychological problems.

**Table 6:** Top 20 keywords with frequency.

Rank	Keywords	Occurrences	Total link strength
1	Obesity	1,543	10,126
2	Overweight	752	5,438
3	Depression	736	5,224
4	BMI	545	3,646
5	Bariatric surgery	499	3,098
6	Weight loss	488	3,420
7	Stress	442	2,922
8	Health	421	2,933
9	Physical activity	404	2,794
10	Risk-factors	384	2,697
11	Health-related quality of life	360	2,612
12	Adolescents	347	2,541
13	Prevalence	323	2,353
14	Children	310	2,188
15	Binge eating disorder	276	1,989
16	Behaviors	261	1,866
17	Weight	258	1,792
18	Association	249	1,827
19	Anxiety	241	1,747
20	Women	218	1,561

**Figure 9:**
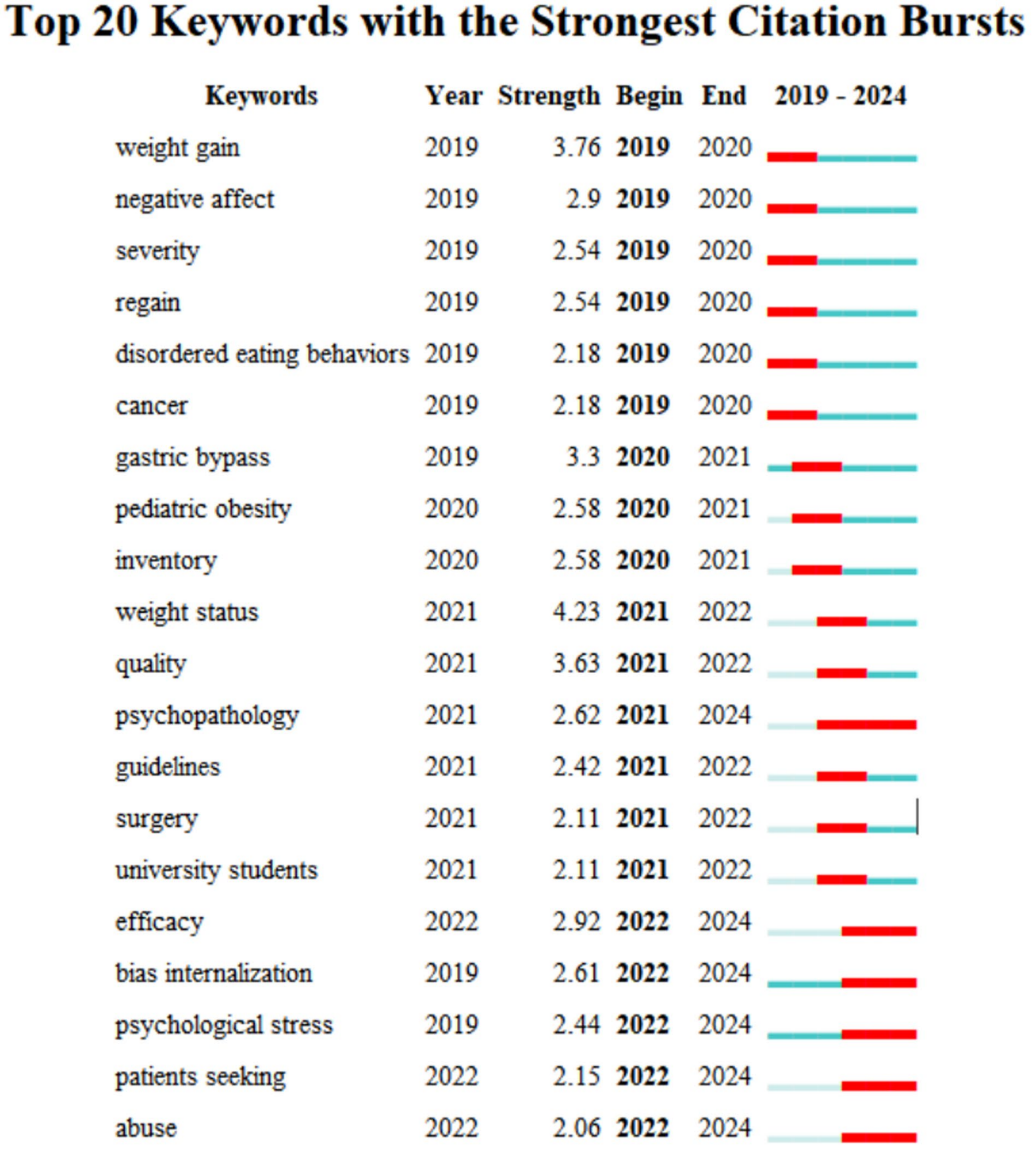
Keyword burst detection of 2019 to 2024.

To further explore the future trends in obesity and mental health, CiteSpace software was utilized to analyze the explosive words in this field over the past 5 years (Figure 9). The results showed that the key areas that current research continued to concentrate on were “psychopathology,” “bias internalization,” “psychological stress,” and “patients seeking.” This suggests that researchers are increasingly inclined to explore the individualized mental health needs of the obese population, offering crucial clues for future research directions.

## 4 Discussion

### 4.1 Research overview

This study employed bibliometric analysis to systematically analyze 2,753 articles in the field of obesity and psychology retrieved from the Web of Science database as of October 20, 2024, utilizing CiteSpace and VOSviewer software. The findings indicate that research in this area commenced in 1975, with a relatively low number of publications before the 21st century. Subsequently, as global obesity issues have become more prominent and their impact on mental health has become more evident, the annual number of publications has been on a continuous upward trend, peaking at 214 articles in 2021. In the past 2 years, there has been a slight decline, and the research enthusiasm has stabilized.

In terms of national distribution, developed countries such as the United States and the United Kingdom play a dominant role in research within this field. As a developing country with remarkable achievements, China has witnessed a substantial increase in the number of published papers. However, there is a significant lack of participation from regions like Africa. Regarding the collaboration among authors and institutions, Yale University, University College London, and other institutions have made outstanding contributions. Nevertheless, the author collaboration network is relatively limited, and cross-regional collaboration is particularly scarce. It is recommended to establish an international cooperation network, enhance academic exchanges, and promote research and development. Journal analysis reveals that journals such as *Obesity Surgery* have the highest publication volume, but there are relatively few high-impact-factor and OA journals.

Given the insufficient interdisciplinary collaboration in the field of obesity psychology, it is essential to establish a fair global research partnership to address this deficiency. Specific measures to promote fair cooperation include: developed countries should proactively share their research data, technology, and platforms, offer technical support and training to developing countries, and bridge the gap in research capabilities; encourage the formation of cross-cultural research teams, respect and integrate research perspectives from different cultural backgrounds, and ensure that research designs and results are more universally applicable. Additionally, regular international academic conferences, seminars, and training programs can be organized to facilitate continuous exchange and cooperation among global researchers, thus establishing a long-term and sustainable cooperation mechanism.

### 4.2 Research trends and research hotspots

The combination of literature co-citation analysis and high-frequency keyword analysis not only reveals the research hotspots in the field of obesity and psychology but also conducts an in-depth analysis of the paradigm shifts and evolutionary trends of research in this field. This provides a multi-level and multi-dimensional analytical perspective for further exploring the complex relationship between obesity and mental health. These hotspots encompass the prevalence of psychological distress symptoms like depression and stress, the impact of important mental health problems such as binge eating disorder, and the central role of psychosocial factors including health-related quality of life and weight stigma in obesity intervention.

#### 4.2.1 Psychological distress is prevalent in obese people

Over the past four decades, the global prevalence of obesity has witnessed a substantial increase. Data indicates that the proportion of obese girls has soared eight-fold, reaching 5.6%, while the proportion of obese boys has increased 10 times, hitting 7.8% (35). During the same period, the obesity rate among adults also rose significantly, with 14.9% for women and 10.8% for men (36). Emotional disorders and high levels of negative emotions play a key role in the onset and persistence of obesity. A meta-analysis revealed that, when compared to non-obese individuals, obese adults reported a 23–36% increase in self-reported depressive symptoms and a 14–34% increase in clinically diagnosed major depressive disorder (37). Moreover, there is a significant positive correlation between anxiety and obesity (38). A meta-analysis of depressive symptoms in overweight and obese children found that the clinical prevalence of depression in the obese group ranged from 1.7 to 26.7%, whereas in the overweight group, it ranged from 4.0 to 16.9%. In the study of major depressive disorder, the prevalence of obesity in children ranged from 10.1 to 26.7%, and for overweight children, it ranged from 9.0 to 16.9%, both significantly higher than that of the healthy control group (39). Additionally, a cross-sectional study of 4,579 individuals aged 60 and above in China found that 8% reported depressive symptoms, and women were more prone to it. 23% of obese individuals suffered from depression, which increased the risk of obesity by 37% (40). A multitude of cross-sectional studies have confirmed that obesity-related psychological distress is intricately influenced by multiple factors such as age, ethnicity, gender, and the social environment.

#### 4.2.2 Pharmacological strategies for obesity and depression

This study discovered that depression is the most common psychological distress among obese patients, and the relationship between depression and obesity has emerged as a crucial topic in clinical and scientific research. Some antidepressants may lead to weight gain or even obesity, yet their effects vary according to the type of medication and individual differences. For instance, tricyclic antidepressants (such as amitriptyline) and certain selective serotonin reuptake inhibitors (such as paroxetine) are frequently linked to weight gain. This might be attributed to their impacts on appetite regulation, energy metabolism, and the activity of neurotransmitters like serotonin, histamine, and dopamine. Conversely, drugs such as bupropion and duloxetine exhibit weight-neutral or weight-reducing effects, possibly because of their unique inhibitory effects on the reuptake of dopamine and norepinephrine. Moreover, the effects of antidepressants on metabolic indicators such as insulin sensitivity, lipid metabolism, and body fat distribution are also significant in the development of obesity (41, 42). Morriss et al. (43) carried out a survey on 21,436 overweight or obese adults who were taking at least one antidepressant. The results indicated that all types of antidepressants increased the relative risk of cardiovascular disease and diabetes (any type) when compared with non-antidepressant users. With the exception of citalopram, commonly used antidepressants can raise mortality rates. A randomized controlled trial (44) demonstrated that although naltrexone/bupropion cannot reduce binge eating, it is effective in reducing the weight of patients with binge eating disorder. Evidently, there is significant heterogeneity among different antidepressant drugs. Future research should further explore the mechanism of action of antidepressants, optimize individualized treatment strategies, and provide a scientific basis for managing obesity-related risks.

#### 4.2.3 Eating disorders and mental health in obese patients

Disruptive eating behaviors, including binge eating, emotional eating, and night eating syndrome, are highly prevalent among the obese population. These behaviors can give rise to psychological issues such as impaired emotional regulation, depression, and anxiety (45). Simultaneously, mental health issues like low self-esteem, heightened perceived stress, and negative emotional states can further exacerbate eating disorders, thus creating a vicious cycle (27). Research has indicated that weight stigma and sociocultural pressure are significant factors contributing to this association. They not only strengthen negative self-perceptions but may also hinder individuals from seeking professional assistance (29). Therefore, in the psychological health intervention for obese patients, priority should be placed on identifying and treating eating disorders. Meanwhile, integrating psychosocial and social support is essential to disrupt this vicious cycle and promote the overall physical and mental well-being of the patients.

#### 4.2.4 Is bariatric surgery effective in improving psychological distress in severely obese patients?

An increasing amount of research suggests that weight loss surgery holds the potential to alleviate psychological distress. Multiple studies have demonstrated that after undergoing surgery, patients experience a significant reduction in symptoms of depression and anxiety. This improvement is closely associated with weight loss, enhanced metabolic function, and better social functioning (46). Weight loss surgery may enhance mental health by regulating gut hormones and directly influencing the brain regions responsible for emotional regulation. Additionally, weight loss reduces weight-related stigma, boosts patients’ satisfaction with their self-image, and increases their social participation (47, 48). However, the long-term impact of weight loss surgery on psychological distress remains a subject of controversy. Some studies indicate that as time passes, the improvement in psychological state may diminish, and some patients may even experience more severe psychological distress. This could be attributed to poor postoperative adaptation or weight regain (49). A meta-analysis (50) has confirmed that glucagon-like peptide-1 receptor agonists are both highly effective and safe for patients who experience weight rebound after surgery or insufficient weight loss. Nevertheless, given the limited follow-up period, further research is required to determine their long-term clinical benefits.

#### 4.2.5 The role of psychosocial factors in obesity interventions: a health-related quality of life and weight stigma perspective

Psychosocial factors play an important role in obesity intervention, with health-related quality of life and weight stigma standing out as two key perspectives. Obese patients frequently endure psychological distress as a result of weight discrimination and social bias. This distress leads to a significant decrease in their health-related quality of life, which is manifested through symptoms like depression, anxiety, and impaired social functioning. Weight stigma not only takes a toll on patients’ mental health but may also prompt them to adopt unhealthy coping strategies, such as overeating or avoiding social interactions. These behaviors, in turn, can further exacerbate obesity (51). Studies have revealed that effective obesity interventions should focus on both weight management and psychosocial support. For example, cognitive behavioral therapy can be employed to alleviate weight stigma, and group interventions can be conducted to augment social support networks (52). Improving health-related quality of life not only improves patients’ subjective well-being but also enhances their adherence to lifestyle interventions, ultimately leading to better weight loss.

## 5 Conclusion

This study reveals a complex bidirectional association between obesity and psychological factors. On one hand, obese individuals are more prone to experiencing psychological distress, including depression, anxiety, and low self-esteem. These psychological issues are intricately linked to multiple factors, such as social discrimination, dissatisfaction with their body image, and metabolic changes. On the other hand, psychological factors like emotional eating, stress response disorders, and cognitive-behavioral patterns play significant roles in the occurrence and continuous progression of obesity. Moreover, the potential efficacy of psychological interventions, such as cognitive behavioral therapy (53) and mindfulness training (54), in obesity management has been validated. Future research should delve deeper into the biological mechanisms underlying the relationship between obesity and mental health. It is also essential to optimize interdisciplinary intervention strategies and take into account the special needs of different populations, including children, adolescents, and women. By doing so, we can achieve synergistic improvement in obesity conditions and mental health levels.

## 6 Limitations

This study has certain limitations. Firstly, the co-citation analysis and visualization were solely based on data retrieved from the SCI-E database. This approach may limit the representativeness of the research findings because data from other databases cannot be utilized for joint analysis. Secondly, even though a systematic search strategy was employed, some relevant articles might have been missed. This was due to the constraints in search criteria, document types, and language. Nevertheless, the influence of these omissions on the overall results was likely to be limited. Moreover, the analysis in this study did not encompass the research dynamics in a specific field or region (economically underdeveloped areas). This may undermine the generalizability of the research conclusions. Further research can improve the comprehensiveness and representativeness of the study by expanding the database scope, optimizing search strategies, and including multilingual literature.

## Data Availability

The original contributions presented in the study are included in the article/Supplementary material, further inquiries can be directed to the corresponding author.
