# Supplementary material for: Obesity and psychology: a bibliometric analysis of half a century
Source: Front Nutr. 2025 Apr 16;12:1539587. doi: 10.3389/fnut.2025.1539587 (PMC12040703; doi:10.3389/fnut.2025.1539587)
Supplement: Supplementary file 1 [file Table_1.docx]

Supplementary Material

**Obesity and psychology :a bibliometric analysis of half a century**

**Haiqiu Zhou^1^,** **Shan Liu^1^, Zixuan Xiao^1^, Saiqiong Yin^1^, Boyan Fan^1^, Guixiang Sun^1*^**

^1^ College of Traditional Chinese Medicine, Hunan University of Traditional Chinese Medicine, Changsha City, Hunan Province, China

* **Correspondence**:

[003575@hnucm.edu.cn](mailto:003575@hnucm.edu.cn)

# Supplementary Tables

**Supplementary Table 1. Number of publications per year**

| year | 1975 | 1976 | 1977 | 1979 | 1980 | 1981 | 1982 | 1983 | 1984 | 1985 | 1986 | 1987 | 1988 | 1989 | 1990 | 1991 | 1992 | 1993 | 1994 | 1995 | 1996 | 1997 | 1998 | 1999 | 2000 |
| --- | --- | --- | --- | --- | --- | --- | --- | --- | --- | --- | --- | --- | --- | --- | --- | --- | --- | --- | --- | --- | --- | --- | --- | --- | --- |
| NP | 1 | 5 | 5 | 3 | 1 | 2 | 4 | 2 | 5 | 1 | 5 | 3 | 2 | 3 | 2 | 2 | 7 | 6 | 7 | 14 | 9 | 7 | 16 | 14 | 14 |
| year | 2001 | 2002 | 2003 | 2004 | 2005 | 2006 | 2007 | 2008 | 2009 | 2010 | 2011 | 2012 | 2013 | 2014 | 2015 | 2016 | 2017 | 2018 | 2019 | 2020 | 2021 | 2022 | 2023 | 2024 |  |
| NP | 23 | 21 | 20 | 38 | 46 | 52 | 66 | 80 | 78 | 93 | 86 | 96 | 108 | 130 | 157 | 129 | 153 | 164 | 177 | 168 | 214 | 195 | 167 | 152 |  |

**Supplementary Table 2. Top 30 Country Collaboration Network**

| Rank | label | x | y | cluster | weight<Links> | weight<Total link strength> |
| --- | --- | --- | --- | --- | --- | --- |
| 1 | usa | 0.307 | 0.2738 | 2 | 36 | 304 |
| 2 | united kingdom | 0.2487 | -0.0047 | 2 | 39 | 306 |
| 3 | australia | 0.4017 | 0.0177 | 6 | 34 | 154 |
| 4 | italy | -0.4835 | -0.0165 | 1 | 32 | 158 |
| 5 | canada | 0.1315 | -0.0201 | 6 | 29 | 115 |
| 6 | germany | -0.2138 | -0.0604 | 3 | 31 | 149 |
| 7 | netherlands | -0.6459 | 0.2918 | 3 | 22 | 108 |
| 8 | peoples r china | 0.8943 | 0.3514 | 2 | 17 | 68 |
| 9 | spain | -0.6226 | 0.1062 | 3 | 28 | 121 |
| 10 | france | -0.6056 | -0.0227 | 1 | 28 | 92 |
| 11 | sweden | -0.0658 | -0.2499 | 4 | 26 | 107 |
| 12 | brazil | 0.6042 | -0.5207 | 4 | 15 | 51 |
| 13 | iran | 0.6308 | 0.2738 | 6 | 8 | 32 |
| 14 | portugal | -0.1363 | 0.0598 | 4 | 20 | 51 |
| 15 | poland | -0.7613 | -0.075 | 1 | 12 | 16 |
| 16 | switzerland | 0.0829 | -0.5845 | 5 | 22 | 69 |
| 17 | finland | 0.2944 | -0.2359 | 4 | 16 | 45 |
| 18 | turkey | -0.2592 | -0.6746 | 1 | 10 | 19 |
| 19 | norway | 0.2598 | -0.7043 | 4 | 16 | 37 |
| 20 | south korea | 0.9841 | 0.8033 | 2 | 3 | 8 |
| 21 | belgium | -0.8613 | 0.0593 | 1 | 26 | 70 |
| 22 | denmark | -0.304 | 0.1566 | 1 | 17 | 45 |
| 23 | ireland | -0.944 | -0.1717 | 1 | 16 | 41 |
| 24 | israel | -0.4906 | -0.085 | 3 | 24 | 62 |
| 25 | japan | 0.9811 | 0.6301 | 2 | 4 | 6 |
| 26 | greece | 0.5326 | -0.0726 | 2 | 15 | 25 |
| 27 | mexico | -0.7632 | 0.4348 | 3 | 16 | 22 |
| 28 | austria | -0.1598 | -0.4724 | 1 | 20 | 37 |
| 29 | india | 0.6858 | -0.6332 | 5 | 11 | 13 |
| 30 | new zealand | -0.5981 | 0.5876 | 3 | 15 | 22 |

**Supplementary Table 3. Top 10 countries annual publications**

| Year | USA | UK | AUSTRALIA | ITALY | CANADA | GERMANY | NETHERLANDS | CHINA | SPAIN | FRANCE |
| --- | --- | --- | --- | --- | --- | --- | --- | --- | --- | --- |
| 1976 | 1 | 0 | 0 | 0 | 0 | 0 | 0 | 0 | 0 | 0 |
| 1977 | 6 | 0 | 0 | 0 | 1 | 0 | 0 | 0 | 0 | 0 |
| 1979 | 11 | 0 | 0 | 0 | 1 | 0 | 0 | 0 | 0 | 0 |
| 1980 | 13 | 0 | 0 | 0 | 1 | 0 | 0 | 0 | 0 | 0 |
| 1981 | 14 | 2 | 0 | 0 | 1 | 0 | 0 | 0 | 0 | 0 |
| 1982 | 17 | 4 | 0 | 0 | 1 | 0 | 0 | 0 | 0 | 0 |
| 1983 | 19 | 4 | 0 | 0 | 5 | 0 | 0 | 0 | 0 | 0 |
| 1984 | 22 | 4 | 0 | 0 | 5 | 0 | 0 | 0 | 0 | 0 |
| 1985 | 23 | 4 | 0 | 0 | 5 | 0 | 0 | 0 | 0 | 0 |
| 1986 | 29 | 4 | 1 | 0 | 5 | 0 | 0 | 0 | 0 | 0 |
| 1987 | 29 | 4 | 2 | 1 | 5 | 0 | 0 | 0 | 0 | 0 |
| 1988 | 30 | 4 | 2 | 1 | 7 | 0 | 0 | 0 | 0 | 0 |
| 1989 | 31 | 4 | 2 | 1 | 7 | 0 | 0 | 0 | 0 | 0 |
| 1990 | 32 | 4 | 2 | 1 | 7 | 0 | 0 | 0 | 0 | 0 |
| 1991 | 33 | 5 | 2 | 1 | 7 | 0 | 0 | 0 | 0 | 0 |
| 1992 | 38 | 6 | 2 | 2 | 7 | 0 | 0 | 0 | 0 | 0 |
| 1993 | 41 | 6 | 2 | 2 | 8 | 0 | 0 | 0 | 0 | 0 |
| 1994 | 48 | 6 | 2 | 2 | 8 | 0 | 0 | 0 | 0 | 0 |
| 1995 | 58 | 9 | 2 | 4 | 8 | 0 | 0 | 0 | 0 | 0 |
| 1996 | 60 | 10 | 8 | 6 | 8 | 0 | 0 | 0 | 0 | 0 |
| 1997 | 64 | 10 | 8 | 6 | 8 | 0 | 4 | 0 | 1 | 0 |
| 1998 | 83 | 12 | 10 | 6 | 8 | 0 | 6 | 0 | 1 | 0 |
| 1999 | 102 | 19 | 11 | 6 | 10 | 0 | 6 | 0 | 1 | 1 |
| 2000 | 118 | 26 | 11 | 8 | 10 | 3 | 8 | 0 | 1 | 1 |
| 2001 | 143 | 28 | 13 | 13 | 11 | 6 | 8 | 0 | 1 | 1 |
| 2002 | 169 | 34 | 14 | 18 | 11 | 11 | 10 | 0 | 6 | 1 |
| 2003 | 184 | 38 | 14 | 19 | 12 | 18 | 18 | 1 | 6 | 7 |
| 2004 | 219 | 44 | 14 | 38 | 18 | 26 | 21 | 1 | 7 | 15 |
| 2005 | 274 | 53 | 16 | 47 | 20 | 31 | 33 | 3 | 7 | 21 |
| 2006 | 325 | 69 | 29 | 53 | 21 | 40 | 37 | 3 | 7 | 22 |
| 2007 | 390 | 90 | 51 | 78 | 33 | 49 | 40 | 3 | 11 | 24 |
| 2008 | 461 | 103 | 71 | 101 | 42 | 69 | 51 | 10 | 14 | 25 |
| 2009 | 523 | 113 | 97 | 127 | 50 | 83 | 68 | 12 | 17 | 29 |
| 2010 | 634 | 124 | 114 | 141 | 72 | 91 | 78 | 23 | 21 | 37 |
| 2011 | 736 | 132 | 132 | 161 | 95 | 94 | 93 | 31 | 26 | 55 |
| 2012 | 854 | 154 | 150 | 168 | 110 | 111 | 114 | 39 | 28 | 65 |
| 2013 | 963 | 184 | 195 | 184 | 126 | 127 | 131 | 48 | 45 | 67 |
| 2014 | 1112 | 210 | 220 | 222 | 141 | 141 | 145 | 56 | 56 | 75 |
| 2015 | 1329 | 239 | 242 | 242 | 176 | 173 | 168 | 67 | 64 | 83 |
| 2016 | 1492 | 261 | 278 | 262 | 201 | 194 | 182 | 74 | 109 | 91 |
| 2017 | 1665 | 295 | 339 | 314 | 227 | 227 | 222 | 83 | 126 | 107 |
| 2018 | 1842 | 343 | 389 | 332 | 267 | 247 | 240 | 103 | 157 | 134 |
| 2019 | 2035 | 399 | 435 | 385 | 303 | 264 | 258 | 116 | 176 | 163 |
| 2020 | 2235 | 471 | 475 | 422 | 340 | 289 | 261 | 152 | 192 | 175 |
| 2021 | 2426 | 515 | 549 | 503 | 378 | 326 | 291 | 189 | 251 | 206 |
| 2022 | 2562 | 594 | 637 | 589 | 432 | 351 | 311 | 247 | 268 | 237 |
| 2023 | 2763 | 628 | 673 | 644 | 454 | 377 | 341 | 310 | 289 | 261 |
| 2024 | 2878 | 669 | 691 | 753 | 462 | 428 | 358 | 389 | 359 | 284 |

**Supplementary Table 4. Co-occurrence cluster analysis of top 30 high-output academic institutions**

| Rank | label | x | y | cluster | Weight  <Links> | Weight  <Total link strength> | Weight  <Documents> |
| --- | --- | --- | --- | --- | --- | --- | --- |
| 1 | Yale Univ | 0.6842 | -0.0214 | 67 | 63 | 82 | 69 |
| 2 | Ucl | -0.3865 | 0.1235 | 31 | 82 | 108 | 52 |
| 3 | Univ Penn | 0.6955 | -0.1499 | 50 | 89 | 112 | 41 |
| 4 | Monash Univ | 0.1561 | 0.0974 | 43 | 84 | 113 | 40 |
| 5 | Columbia Univ | 0.3021 | -0.005 | 30 | 97 | 137 | 39 |
| 6 | Univ Minnesota | 0.7019 | -0.0784 | 2 | 86 | 128 | 39 |
| 7 | Kings Coll London | -0.2545 | 0.0176 | 60 | 125 | 152 | 37 |
| 8 | Univ Toronto | 0.5189 | 0.2188 | 48 | 72 | 121 | 36 |
| 9 | Univ Leipzig | -0.2535 | 0.4564 | 66 | 74 | 110 | 35 |
| 10 | Deakin Univ | 0.1446 | -0.2261 | 59 | 45 | 59 | 34 |
| 11 | Brown Univ | 0.7254 | -0.0329 | 70 | 81 | 126 | 32 |
| 12 | Univ Calif San Francisco | 0.6289 | -0.119 | 35 | 70 | 96 | 32 |
| 13 | Univ Sydney | 0.236 | -0.1444 | 18 | 61 | 96 | 32 |
| 14 | Karolinska Inst | -0.2397 | -0.1759 | 49 | 114 | 146 | 29 |
| 15 | Univ Pittsburgh | 0.4579 | 0.1246 | 9 | 47 | 60 | 29 |
| 16 | Univ Florida | 0.7018 | -0.1822 | 68 | 54 | 74 | 27 |
| 17 | Univ Helsinki | -0.4449 | -0.1364 | 8 | 60 | 103 | 27 |
| 18 | Univ n Carolina | 0.4731 | 0.0471 | 74 | 83 | 92 | 27 |
| 19 | Maastricht Univ | -0.4245 | 0.1644 | 1 | 77 | 92 | 26 |
| 20 | Univ Calif San Diego | 0.607 | -0.0546 | 2 | 72 | 102 | 26 |
| 21 | Univ Leeds | 0.0321 | 0.4152 | 62 | 49 | 70 | 26 |
| 22 | Univ Melbourne | 0.2285 | -0.0651 | 59 | 57 | 91 | 26 |
| 23 | Vrije Univ Amsterdam | -0.3398 | 0.26 | 1 | 59 | 93 | 26 |
| 24 | Harvard Univ | 0.3923 | 0.0768 | 25 | 65 | 79 | 25 |
| 25 | Ohio State Univ | 0.8148 | 0.1303 | 2 | 22 | 28 | 25 |
| 26 | Univ Padua | -0.3681 | -0.1767 | 29 | 66 | 90 | 24 |
| 27 | Harvard Med Sch | 0.6563 | 0.0469 | 25 | 73 | 105 | 23 |
| 28 | Kent State Univ | 0.5528 | -0.0933 | 46 | 27 | 41 | 23 |
| 29 | Univ Alabama Birmingham | 0.8052 | -0.2366 | 61 | 46 | 55 | 23 |
| 30 | Washington Univ | 0.7071 | -0.0452 | 9 | 75 | 104 | 23 |

**Supplementary Table 5. Top 30 authors with the total link strength in the co-occurrence cluster analysis of authors**

| Rank | Label | x | y | Cluster | Weight  <Links> | Weight  <Total Link Strength> | Weight  <Documents> |
| --- | --- | --- | --- | --- | --- | --- | --- |
| 1 | Grilo, Carlos m. | 0.4691 | 0.0391 | 13 | 6 | 22 | 24 |
| 2 | Hay, Phillipa | -1 | -0.0887 | 4 | 12 | 26 | 20 |
| 3 | Heinberg, Leslie j. | 0.3092 | 0.365 | 8 | 5 | 33 | 17 |
| 4 | Wilfley, Denise e. | -0.1085 | -0.1945 | 2 | 11 | 26 | 16 |
| 5 | Hilbert, Anja | -0.5104 | 0.2416 | 3 | 4 | 11 | 14 |
| 6 | Puhl, Rebecca m. | 0.979 | -0.0165 | 1 | 3 | 6 | 14 |
| 7 | Zipfel, Stephan | -0.6392 | 0.0782 | 3 | 12 | 34 | 13 |
| 8 | Ashton, Kathleen | 0.3561 | 0.4282 | 8 | 4 | 27 | 12 |
| 9 | Penninx, Brenda w. j. h. | -0.7499 | -0.3496 | 14 | 3 | 13 | 12 |
| 10 | Crosby, Ross d. | -0.1519 | 0.0735 | 2 | 14 | 22 | 11 |
| 11 | Latner, Janet d. | 0.8376 | 0.0029 | 1 | 7 | 13 | 11 |
| 12 | Lin, Chung-Ying | 0.8371 | 0.0599 | 1 | 3 | 11 | 11 |
| 13 | Marek, Ryan j. | 0.3819 | 0.3452 | 8 | 5 | 27 | 11 |
| 14 | Tanofsky-Kraff, Marian | -0.2587 | -0.2063 | 7 | 6 | 24 | 11 |
| 15 | Ben-Porath, Yossef s. | 0.412 | 0.4041 | 8 | 4 | 25 | 10 |
| 16 | Butryn, Meghan l. | -0.1371 | 0.3159 | 10 | 5 | 22 | 10 |
| 17 | Masheb, Robin m. | 0.5543 | 0.0532 | 13 | 3 | 18 | 10 |
| 18 | Sarwer, David b. | -0.1781 | 0.5952 | 9 | 5 | 14 | 10 |
| 19 | Teufel, Martin | -0.6727 | 0.1389 | 3 | 6 | 21 | 10 |
| 20 | Wing, Rena r. | 0.055 | -0.342 | 11 | 4 | 9 | 10 |
| 21 | Brennan, Leah | -1.0937 | 0.0224 | 4 | 2 | 2 | 9 |
| 22 | Bulik, Cynthia m. | -0.5257 | -0.0726 | 5 | 8 | 11 | 9 |
| 23 | Janicke, David m. | -0.0836 | 0.3271 | 10 | 2 | 3 | 9 |
| 24 | Neumark-Sztainer, Dianne | -0.0182 | -0.2034 | 2 | 1 | 2 | 9 |
| 25 | Robinson, Eric | 1.3235 | -0.1587 | 6 | 5 | 12 | 9 |
| 26 | Tomiyama, a. Janet | 1.1332 | -0.0332 | 1 | 4 | 5 | 9 |
| 27 | White, Marney a. | 0.5476 | 0.0022 | 13 | 3 | 18 | 9 |
| 28 | Boutelle, Kerri n. | 0.0143 | -0.153 | 2 | 4 | 8 | 8 |
| 29 | Epel, Elissa | 1.2307 | 0.069 | 1 | 2 | 5 | 8 |
| 30 | Epel, Elissa s. | 1.1585 | 0.0129 | 1 | 3 | 7 | 8 |

**Supplementary Table 6. Top 20 author annual publications**

| Year | Grilo Cm | Masheb Rm | Hay p | Castelnuovo g | Wilfley De | Wadden Ta | Heinberg Lj | Puhl Rm | Penninx Bwjh | Sarwer Db | Wing Rr | Annesi Jj | Hilbert a | Braet c | Epstein Lh | Sockalingam s | White Ma | Zipfel s | Ashton k | Brownell Kd |
| --- | --- | --- | --- | --- | --- | --- | --- | --- | --- | --- | --- | --- | --- | --- | --- | --- | --- | --- | --- | --- |
| 1984 | 0 | 0 | 0 | 0 | 0 | 1 | 0 | 0 | 0 | 0 | 0 | 0 | 0 | 0 | 0 | 0 | 0 | 0 | 0 | 3 |
| 1985 | 0 | 0 | 0 | 0 | 0 | 1 | 0 | 0 | 0 | 0 | 0 | 0 | 0 | 0 | 0 | 0 | 0 | 0 | 0 | 0 |
| 1992 | 0 | 0 | 0 | 0 | 0 | 2 | 0 | 0 | 0 | 0 | 0 | 0 | 0 | 0 | 0 | 0 | 0 | 0 | 0 | 0 |
| 1993 | 0 | 0 | 0 | 0 | 0 | 0 | 0 | 0 | 0 | 0 | 0 | 0 | 0 | 0 | 0 | 0 | 0 | 0 | 0 | 0 |
| 1994 | 0 | 0 | 0 | 0 | 0 | 0 | 0 | 0 | 0 | 0 | 1 | 0 | 0 | 0 | 1 | 0 | 0 | 0 | 0 | 0 |
| 1995 | 0 | 0 | 0 | 0 | 1 | 0 | 0 | 0 | 0 | 0 | 0 | 0 | 0 | 0 | 0 | 0 | 0 | 0 | 0 | 3 |
| 1996 | 0 | 0 | 0 | 0 | 0 | 0 | 0 | 0 | 0 | 0 | 0 | 0 | 0 | 0 | 1 | 0 | 0 | 0 | 0 | 0 |
| 1997 | 0 | 0 | 0 | 0 | 1 | 1 | 0 | 0 | 0 | 1 | 0 | 0 | 0 | 0 | 0 | 0 | 0 | 0 | 0 | 0 |
| 1998 | 0 | 0 | 0 | 0 | 0 | 1 | 0 | 0 | 0 | 0 | 1 | 0 | 0 | 0 | 2 | 0 | 0 | 0 | 0 | 1 |
| 1999 | 0 | 0 | 0 | 0 | 0 |  | 0 | 0 | 0 | 0 | 1 | 0 | 0 | 0 | 0 | 0 | 0 | 0 | 0 | 0 |
| 2000 | 2 | 2 | 0 | 0 | 0 | 1 | 0 | 0 | 0 | 0 | 1 | 0 | 0 | 0 | 1 | 0 | 0 | 0 | 0 | 0 |
| 2001 | 1 | 1 | 0 | 0 | 0 | 1 | 0 | 0 | 0 | 1 | 0 | 0 | 0 | 0 | 0 | 0 | 0 | 0 | 0 | 0 |
| 2002 | 0 | 0 | 0 | 0 | 1 | 0 | 0 | 0 | 0 | 0 | 0 | 0 | 0 | 0 | 0 | 0 | 0 | 0 | 0 | 0 |
| 2003 | 1 | 1 | 0 | 0 | 0 | 0 | 0 | 0 | 0 | 0 | 0 | 0 | 0 | 1 | 0 | 0 | 0 | 0 | 0 | 0 |
| 2004 | 1 | 1 | 0 | 0 | 0 | 2 | 0 | 0 | 0 | 1 | 0 | 0 | 0 | 0 | 0 | 0 | 0 | 0 | 0 | 0 |
| 2005 | 4 | 4 | 0 | 0 | 0 | 1 | 0 | 1 | 0 | 1 | 0 | 0 | 0 | 1 | 0 | 0 | 0 | 0 | 0 | 1 |
| 2006 | 3 | 1 | 0 | 0 | 1 | 3 | 0 | 1 | 0 | 2 | 0 | 0 | 0 | 2 | 0 | 0 | 2 | 0 | 0 | 1 |
| 2007 | 3 | 2 | 1 | 0 | 0 | 1 | 0 | 1 | 0 | 1 | 0 | 0 | 0 | 0 | 1 | 0 | 2 | 0 | 0 | 0 |
| 2008 | 1 | 1 | 0 | 0 | 0 | 1 | 0 | 0 | 0 | 3 | 0 | 0 | 2 | 1 | 0 | 0 | 1 | 0 | 0 | 0 |
| 2009 | 1 | 1 | 0 | 1 | 0 | 0 | 1 | 0 | 1 | 0 | 1 | 0 | 0 | 2 | 0 | 0 | 1 | 0 | 0 | 1 |
| 2010 | 3 | 3 | 0 | 0 | 2 | 0 | 1 | 2 | 0 | 0 | 0 | 0 | 0 | 0 | 1 | 0 | 3 | 0 | 2 | 0 |
| 2011 | 0 | 0 | 0 | 2 | 0 | 1 | 0 | 0 | 0 | 0 | 0 | 1 | 0 | 0 | 0 | 1 | 0 | 0 | 0 | 0 |
| 2012 | 3 | 3 | 0 | 0 | 1 | 0 | 1 | 1 | 1 | 0 | 0 | 0 | 1 | 1 | 1 | 0 | 3 | 2 | 2 | 1 |
| 2013 | 2 | 1 | 1 | 0 | 1 | 0 | 1 | 2 | 0 | 1 | 0 | 0 | 1 | 0 | 0 | 1 | 1 | 1 | 1 | 0 |
| 2014 | 0 | 0 | 1 | 0 | 0 | 0 | 1 | 1 | 0 | 0 | 2 | 0 | 2 | 1 | 0 | 1 | 0 | 0 | 1 | 1 |
| 2015 | 1 | 0 | 1 | 1 | 0 | 0 | 2 | 0 | 0 | 0 | 2 | 3 | 1 | 0 | 0 | 1 | 0 | 1 | 3 | 0 |
| 2016 | 2 | 1 | 0 | 0 | 4 | 1 | 2 | 1 | 1 | 2 | 0 | 1 | 1 | 0 | 1 | 0 | 0 | 2 | 1 | 0 |
| 2017 | 0 | 1 | 3 | 0 | 2 | 0 | 4 | 0 | 3 | 0 | 0 | 2 | 1 | 0 | 2 | 1 | 0 | 1 | 2 | 0 |
| 2018 | 0 | 0 | 2 | 1 | 2 | 0 | 0 | 1 | 2 | 0 | 0 | 0 | 0 | 0 | 0 | 1 | 0 | 0 | 0 | 0 |
| 2019 | 1 | 0 | 0 | 0 | 1 | 1 | 1 | 2 | 2 | 2 | 2 | 0 | 0 | 0 | 1 | 2 | 0 | 0 | 0 | 0 |
| 2020 | 0 | 0 | 1 | 0 | 0 | 0 | 0 | 3 | 1 | 0 | 0 | 1 | 0 | 1 | 0 | 2 | 0 | 0 | 0 | 0 |
| 2021 | 2 | 0 | 6 | 5 | 1 | 0 | 3 | 0 | 2 | 0 | 1 | 3 | 1 | 1 | 0 | 1 | 0 | 3 | 0 | 0 |
| 2022 | 1 | 0 | 3 | 3 | 0 | 0 | 0 | 1 | 1 | 0 | 2 | 1 | 0 | 1 | 0 | 2 | 0 | 2 | 0 | 0 |
| 2023 | 3 | 0 | 1 | 1 | 2 | 0 | 0 | 0 | 1 | 0 | 1 | 1 | 2 | 1 | 1 | 0 | 0 | 0 | 0 | 0 |
| 2024 | 2 | 0 | 1 | 6 | 0 | 0 | 0 | 0 | 1 | 0 | 0 | 1 | 2 | 0 | 0 | 0 | 0 | 1 | 0 | 0 |

**Supplementary Table 7. Top 30 highly cited articles**

| **Paper** | **DOI** | **Total Citations** | **TC per Year** | **Normalized TC** |
| --- | --- | --- | --- | --- |
| SLAVICH GM, 2014, PSYCHOL BULL | 10.1037/a0035302 | 1310 | 109.17 | 20.57 |
| PUHL RM, 2010, AM J PUBLIC HEALTH | 10.2105/AJPH.2009.159491 | 1180 | 73.75 | 13.47 |
| ADAM TC, 2007, PHYSIOL BEHAV | 10.1016/j.physbeh.2007.04.011 | 1137 | 59.84 | 12.76 |
| PUHL RM, 2007, PSYCHOL BULL | 10.1037/0033-2909.133.4.557 | 884 | 46.53 | 9.92 |
| PARSONS TJ, 1999, INT J OBESITY | NA | 836 | 30.96 | 6.75 |
| PHELAN SM, 2015, OBES REV | 10.1111/obr.12266 | 756 | 68.73 | 15.93 |
| EPEL E, 2001, PSYCHONEUROENDOCRINO | 10.1016/S0306-4530(00)00035-4 | 725 | 29.00 | 6.86 |
| STANGL AL, 2019, BMC MED | 10.1186/s12916-019-1271-3 | 713 | 101.86 | 21.08 |
| PUHL RM, 2006, OBESITY | 10.1038/oby.2006.208 | 654 | 32.70 | 7.18 |
| GREENO CG, 1994, PSYCHOL BULL | 10.1037/0033-2909.115.3.444 | 615 | 19.22 | 4.55 |
| JANSSEN I, 2004, PEDIATRICS | 10.1542/peds.113.5.1187 | 606 | 27.55 | 6.11 |
| DAVIS CL, 2011, HEALTH PSYCHOL | 10.1037/a0021766 | 583 | 38.87 | 11.89 |
| FRIEDMAN MA, 1995, PSYCHOL BULL | 10.1037/0033-2909.117.1.3 | 561 | 18.10 | 5.35 |
| DI CESARE M, 2019, BMC MED | 10.1186/s12916-019-1449-8 | 561 | 80.14 | 16.58 |
| DIXON JB, 2010, MOL CELL ENDOCRINOL | 10.1016/j.mce.2009.07.008 | 558 | 34.88 | 6.37 |
| DE WIT L, 2010, PSYCHIAT RES | 10.1016/j.psychres.2009.04.015 | 534 | 33.38 | 6.10 |
| GARIEPY G, 2010, INT J OBESITY | 10.1038/ijo.2009.252 | 487 | 30.44 | 5.56 |
| RUBINO F, 2020, NAT MED | 10.1038/s41591-020-0803-x | 484 | 80.67 | 20.03 |
| GIBSON EL, 2006, PHYSIOL BEHAV | 10.1016/j.physbeh.2006.01.024 | 480 | 24.00 | 5.27 |
| PULGARÓN ER, 2013, CLIN THER | 10.1016/j.clinthera.2012.12.014 | 436 | 33.54 | 7.11 |
| TEIXEIRA PJ, 2015, BMC MED | 10.1186/s12916-015-0323-6 | 425 | 38.64 | 8.96 |
| CAMPBELL JA, 2016, AM J PREV MED | 10.1016/j.amepre.2015.07.022 | 423 | 42.30 | 10.84 |
| EPSTEIN LH, 1998, PEDIATRICS | NA | 420 | 15.00 | 6.11 |
| TOMIYAMA AJ, 2014, APPETITE | 10.1016/j.appet.2014.06.108 | 417 | 34.75 | 6.55 |
| YAU YHC, 2013, MINERVA ENDOCRINOL | NA | 404 | 31.08 | 6.58 |
| MACLEAN PS, 2015, OBESITY | 10.1002/oby.20967 | 397 | 36.09 | 8.37 |
| WILFLEY DE, 2002, ARCH GEN PSYCHIAT | 10.1001/archpsyc.59.8.713 | 396 | 16.50 | 4.47 |
| SINHA R, 2013, BIOL PSYCHIAT | 10.1016/j.biopsych.2013.01.032 | 386 | 29.69 | 6.29 |
| GRIFFITHS LJ, 2010, INT J PEDIATR OBES | 10.3109/17477160903473697 | 379 | 23.69 | 4.33 |
| TOMIYAMA AJ, 2019, ANNU REV PSYCHOL | 10.1146/annurev-psych-010418-102936 | 378 | 54.00 | 11.17 |

**Supplementary Table 8. Top 30 highly co-cited articles**

| Rank | Count | Centrality | Burst | Year | Cited Ref |
| --- | --- | --- | --- | --- | --- |
| 1 | 70 | 0.3 | 19.91 | 2011 | Mittal Va, 2011, Psychiat Res, v189, p158, Doi 10.1016/j.Psychres.2011.06.006 |
| 2 | 51 | 0.01 | 25.26 | 2010 | Luppino Fs, 2010, Arch Gen Psychiat, v67, p220, Doi 10.1001/Archgenpsychiatry.2010.2 |
| 3 | 43 | 0.03 | 19.92 | 2014 | Carter Mj, 2014, Ther Recreat j, v48, p275 |
| 4 | 39 | 0.01 | 20.89 | 2006 | Ogden Cl, 2006, Jama-j Am Med Assoc, v295, p1549, Doi 10.1001/Jama.295.13.1549 |
| 5 | 36 | 0 | 16.67 | 2014 | Ng m, 2014, Lancet, v384, p766, Doi 10.1016/s0140-6736(14)60460-8 |
| 6 | 35 | 0 | 15.8 | 2017 | Ezzati m, 2017, Lancet, v390, p2627, Doi 10.1016/s0140-6736(17)32129-3 |
| 7 | 34 | 0.01 | 15.74 | 2014 | Ogden Cl, 2014, Jama-j Am Med Assoc, v311, p806, Doi 10.1001/Jama.2014.732 |
| 8 | 33 | 0.07 | 16.96 | 2009 | Puhl Rm, 2009, Obesity, v17, p941, Doi 10.1038/Oby.2008.636 |
| 9 | 32 | 0.05 | 16.18 | 2013 | Kessler Rc, 2013, Biol Psychiat, v73, p904, Doi 10.1016/j.Biopsych.2012.11.020 |
| 10 | 29 | 0.02 | 14.15 | 2018 | Wu Yk, 2018, j Adv Nurs, v74, p1030, Doi 10.1111/Jan.13511 |
| 11 | 28 | 0.01 | 13.66 | 2018 | Pearl Rl, 2018, Obes Rev, v19, p1141, Doi 10.1111/Obr.12701 |
| 12 | 27 | 0.02 | 13.52 | 2006 | Simon Ge, 2006, Arch Gen Psychiat, v63, p824, Doi 10.1001/Archpsyc.63.7.824 |
| 13 | 26 | 0.11 | 12.35 | 2012 | Bauchowitz Au, 2005, Psychosom Med, v67, p825, Doi 10.1097/01.Psy.0000174173.32271.01 |
| 14 | 26 | 0 | 13.09 | 2005 | Livhits m, 2012, Obes Surg, v22, p70, Doi 10.1007/s11695-011-0472-4 |
| 15 | 25 | 0.01 | 13.75 | 2011 | Flegal Km, 2010, Jama-j Am Med Assoc, v303, p235, Doi 10.1001/Jama.2009.2014 |
| 16 | 25 | 0.01 | 13.95 | 2010 | De Zwaan m, 2011, j Affect Disorders, v133, p61, Doi 10.1016/j.Jad.2011.03.025 |
| 17 | 24 | 0.02 | 12.01 | 2006 | Dawes Aj, 2016, Jama-j Am Med Assoc, v315, p150, Doi 10.1001/Jama.2015.18118 |
| 18 | 24 | 0.02 | 11.21 | 2007 | Fabricatore An, 2006, Obes Surg, v16, p567, Doi 10.1381/096089206776944986 |
| 19 | 24 | 0.08 | 10.94 | 2016 | Kalarchian Ma, 2007, Am j Psychiat, v164, p328, Doi 10.1176/Appi.Ajp.164.2.328 |
| 20 | 23 | 0 | 12.71 | 2003 | Onyike Cu, 2003, Am j Epidemiol, v158, p1139, Doi 10.1093/Aje/Kwg275 |
| 21 | 22 | 0.02 | 11.28 | 2009 | Oude Luttikhuis h, 2009, Cochrane Db Syst Rev, v0, p0, Doi 10.1002/14651858.Cd001872.Pub2 |
| 22 | 21 | 0.01 | 11.6 | 2004 | Meany g, 2014, Eur Eat Disord Rev, v22, p87, Doi 10.1002/Erv.2273 |
| 23 | 21 | 0.05 | 9.69 | 2014 | Rankin j, 2016, Adolesc Health Med t, v7, p125, Doi 10.2147/Ahmt.s101631 |
| 24 | 21 | 0.01 | 9.69 | 2014 | Sarwer Db, 2004, Obes Surg, v14, p1148, Doi 10.1381/0960892042386922 |
| 25 | 21 | 0 | 11.42 | 2016 | Tomiyama Aj, 2014, Appetite, v82, p8, Doi 10.1016/j.Appet.2014.06.108 |
| 26 | 20 | 0.01 | 10.06 | 2005 | Flegal Km, 2002, Jama-j Am Med Assoc, v288, p1723, Doi 10.1001/Jama.288.14.1723 |
| 27 | 20 | 0 | 10.67 | 2006 | Hales Craig m, 2020, Nchs Data Brief, v0, p1 |
| 28 | 20 | 0.01 | 11.59 | 2002 | Heo m, 2006, Int j Obesity, v30, p513, Doi 10.1038/Sj.Ijo.0803122 |
| 29 | 20 | 0.01 | 8.98 | 2020 | Van Hout Gcm, 2005, Obes Surg, v15, p552, Doi 10.1381/0960892053723484 |
| 30 | 19 | 0.02 | 8.65 | 2016 | Puhl Rm, 2020, Am Psychol, v75, p274, Doi 10.1037/Amp0000538 |

**Supplementary Table 9. Summary of the largest 14 co-cited clusters**

| Cluster ID | Size | Mean  (Year) | Top Terms(LSI) | Top Terms(LLR) | Terms(MI) |
| --- | --- | --- | --- | --- | --- |
| 0 | 226 | 2019 | weight stigma; psychological distress; systematic review; eating disorder; bariatric surgery \| conduct disorder; controlled trial; considering psychological factor; reframing intervention; drink consumption | eating disorder risk (558.48, 1.0E-4); weight stigma scale (546.95, 1.0E-4); covid-19 pandemic (506.63, 1.0E-4); cross-sectional study (499.06, 1.0E-4); weight self-stigma questionnaire (437.57, 1.0E-4) | general practitioner (3.55); eating disorder psychopathology (3.55); beck depression inventory (3.55); african american young adult (3.55); group program (3.55) |
| 1 | 190 | 2006 | depressive symptom; body mass index; systematic review; psychological distress; obese children \| health-related quality; weight loss; general population; weight bia; psychological complication | depressive symptom (780.83, 1.0E-4); weight stigma (596.69, 1.0E-4); body mass index (565.68, 1.0E-4); eating disorder (526.36, 1.0E-4); obese children (497.55, 1.0E-4) | self-reported weight status (1.53); mainland chinese adolescent (1.53); general practitioner (1.53); eating disorder psychopathology (1.53); beck depression inventory (1.53) |
| 2 | 129 | 2014 | weight stigma; systematic review; psychological distress; psychological well-being; mediating role \| health-related quality; african american young adult; urban sample; body mass index; maladaptive coping responses | weight stigma (890.88, 1.0E-4); eating disorder (417, 1.0E-4); bariatric surgery (302.33, 1.0E-4); weight disorder (298.35, 1.0E-4); psychological mediation framework-a (294.51, 1.0E-4) | african american young adult (1.18); urban sample (1.18); general practitioner (1.18); eating disorder psychopathology (1.18); beck depression inventory (1.18) |
| 3 | 126 | 2003 | bariatric surgery; following bariatric surgery; weight loss surgery; psychosocial predictor; prognostic significance \| high-risk factor; bariatric surgery patient; postoperative counselling group; behavioral rating system; cleveland clinic | weight loss surgery (381.1, 1.0E-4); psychosocial predictor (350.3, 1.0E-4); prognostic significance (312.35, 1.0E-4); following bariatric surgery (299.72, 1.0E-4); psychosocial evaluation (291.85, 1.0E-4) | general practitioner (0.22); eating disorder psychopathology (0.22); beck depression inventory (0.22); african american young adult (0.22); group program (0.22) |
| 4 | 122 | 2012 | bariatric surgery; bariatric surgery patient; weight loss; systematic review; mental health \| gastric bypass; self-reported eating disorder symptom; super obesity-a 5-year follow-up study; duodenal switch; weight loss surgery candidate | bariatric surgery (1206.34, 1.0E-4); bariatric surgery patient (789.44, 1.0E-4); weight loss (410.07, 1.0E-4); recent research (308.42, 1.0E-4); weight stigma (291.19, 1.0E-4) | beck depression inventory (0.71); bariatric surgical procedure (0.71); general practitioner (0.71); eating disorder psychopathology (0.71); african american young adult (0.71) |
| 5 | 89 | 2010 | eating disorder; psychological treatment; binge-eating disorder; binge eating disorder; obese patient \| art review; incentive salience; other hunger game; eating beliefs questionnaire; binge eating | eating disorder (2256.48, 1.0E-4); psychological treatment (761.62, 1.0E-4); binge-eating disorder (480.68, 1.0E-4); subsyndromal binge (327.48, 1.0E-4); binge eating disorder (326.04, 1.0E-4) | eating disorder psychopathology (0.62); anorexia nervosa (0.62); body composition (0.62); general practitioner (0.62); beck depression inventory (0.62) |
| 6 | 85 | 1995 | eating disorder; eating behavior; weight cycling women; severe binge; psychological symptom \| night eating; disordered eating; binge eating disorder; weight management; psychological treatment | weight cycling women (112.66, 1.0E-4); severe binge (112.66, 1.0E-4); psychological symptom (98.22, 1.0E-4); psychological effect (95.38, 1.0E-4); weight cycling (95.38, 1.0E-4) | bariatric surgery (0.02); weight stigma (0.02); systematic review (0.02); eating disorder (0.02); psychological distress (0.01) |
| 7 | 76 | 1999 | laparoscopic adjustable gastric banding; obesity surgery; psychosocial aspect; appearance orientation; weight loss \| essential role; psychological support; short term; restrictive gastric surgery; obese patient | epidemiology morbidity (182.87, 1.0E-4); appearance orientation (172.66, 1.0E-4); 20-year prospective study (162.46, 1.0E-4); psychosocial effect (142.08, 1.0E-4); laparoscopic adjustable gastric banding (128.22, 1.0E-4) | mental health family function (0.03); african-american women (0.03); general practitioner (0.03); eating disorder psychopathology (0.03); beck depression inventory (0.03) |
| 9 | 62 | 1991 | psychological correlate; next research generation; obese children; psychological problem; socioeconomic status \| low-calorie diet; weight fluctuation; severe obesity; psychological correlate; next research generation | next research generation (74.89, 1.0E-4); maternal psychopathology (59.78, 1.0E-4); psychological problem (59.78, 1.0E-4); low-calorie diet (44.74, 1.0E-4); psychological correlate (42.2, 1.0E-4) | bariatric surgery (0.02); weight stigma (0.02); systematic review (0.02); eating disorder (0.02); psychological distress (0.02) |
| 10 | 60 | 2017 | bariatric surgery; bariatric surgery patient; systematic review; weight outcome; weight loss \| disordered eating; conceptual consideration; patient satisfaction; clinical decision; range effect | bariatric surgery (536.56, 1.0E-4); weight outcome (357.94, 1.0E-4); bariatric surgery patient (300.05, 1.0E-4); cognitive behavioral therapy (243.27, 1.0E-4); postoperative mental health (220.08, 1.0E-4) | personality psychopathology (0.26); longitudinal prediction (0.26); general practitioner (0.26); eating disorder psychopathology (0.26); beck depression inventory (0.26) |
| 13 | 39 | 2016 | food addiction; yale food addiction scale; study protocol; combining cognitive bias modification training; eating disorder \| seeking bariatric surgery; mindful eating; psychological eating style; depressive symptom; psychosocial explanation | food addiction (214.81, 1.0E-4); yale food addiction scale (193.09, 1.0E-4); combining cognitive bias modification training (162.46, 1.0E-4); randomised controlled feasibility trial (162.46, 1.0E-4); physiological trajectory (131.88, 1.0E-4) | socioeconomic disadvantage (0.03); general practitioner (0.03); eating disorder psychopathology (0.03); beck depression inventory (0.03); african american young adult (0.03) |
| 14 | 37 | 1999 | adolescent obesity; psychiatric aspect; research issue; genetic testing; curative intervention \| depressive symptom; body mass index; adolescent obesity; population-based study; behavioral assessment | research issue (115.02, 1.0E-4); genetic testing (115.02, 1.0E-4); adolescent obesity (103.45, 1.0E-4); psychosocial characteristics (102.98, 1.0E-4); curative intervention (102.98, 1.0E-4) | bariatric surgery (0.02); weight stigma (0.02); systematic review (0.02); eating disorder (0.02); psychological distress (0.01) |
| 15 | 27 | 2010 | obese women; emotional eating; systematic review; paediatric obesity; emotional cue \| chronic psychological complaint; obese worker; emotional exhaustion; mood change; potential mechanism | paediatric obesity (185.69, 1.0E-4); weight-management treatments effect (183.1, 1.0E-4); emotional cue (177.51, 1.0E-4); indirect effect (169.33, 1.0E-4); obese women (162.33, 1.0E-4) | general practitioner (0.09); eating disorder psychopathology (0.09); beck depression inventory (0.09); african american young adult (0.09); group program (0.09) |
| 16 | 20 | 2009 | obese pregnant women; depressed mood; mental health; prospective controlled cohort study; gestational weight gain \| pregravid body mass index; psychological factor; breastfeeding duration; early introduction; complementary food | obese pregnant women (233.93, 1.0E-4); pregravid body mass index (166.67, 1.0E-4); prospective controlled cohort study (122.02, 1.0E-4); depressed mood (122.02, 1.0E-4); gestational weight gain (110.88, 1.0E-4) | scottish adult population (0.02); general practitioner (0.02); eating disorder psychopathology (0.02); beck depression inventory (0.02); african american young adult (0.02) |

**Supplementary Table 10. Top 30 highly keywords**

| Rank | Label | x | y | Cluster | Weight  <Links> | Weight  <Total Link Strength> | Weight |
| --- | --- | --- | --- | --- | --- | --- | --- |
| 1 | Obesity | -0.95 | 0.6104 | 1 | 195 | 10126 | 1543 |
| 2 | Overweight | -0.5 | 0.6427 | 3 | 194 | 5438 | 752 |
| 3 | Depression | -0.95 | 0.3047 | 1 | 194 | 5224 | 736 |
| 4 | Bmi | -0.95 | 0.1689 | 1 | 185 | 3646 | 545 |
| 5 | Bariatric Surgery | -0.05 | 0.1143 | 5 | 181 | 3098 | 499 |
| 6 | Weight Loss | -0.05 | 0.9179 | 5 | 187 | 3420 | 488 |
| 7 | Stress | -0.95 | 0.8142 | 1 | 189 | 2922 | 442 |
| 8 | Health | -0.5 | 0.5768 | 3 | 191 | 2933 | 421 |
| 9 | Physical Activity | -0.275 | 0.5964 | 4 | 186 | 2794 | 404 |
| 10 | Risk-Factors | -0.95 | 0.7632 | 1 | 186 | 2697 | 384 |
| 11 | Health-Related Quality Of Life | -0.05 | 0.3071 | 5 | 188 | 2612 | 360 |
| 12 | Adolescents | -0.5 | 0.1159 | 3 | 176 | 2541 | 347 |
| 13 | Prevalence | -0.725 | 0.6676 | 2 | 186 | 2353 | 323 |
| 14 | Children | -0.5 | 0.4012 | 3 | 171 | 2188 | 310 |
| 15 | Binge Eating Disorder | -0.725 | 0.1382 | 2 | 179 | 1989 | 276 |
| 16 | Behaviors | -0.725 | 0.1206 | 2 | 179 | 1866 | 261 |
| 17 | Weight | -0.5 | 0.8622 | 3 | 178 | 1792 | 258 |
| 18 | Association | -0.95 | 0.1179 | 1 | 179 | 1827 | 249 |
| 19 | Anxiety | -0.05 | 0.0821 | 5 | 180 | 1747 | 241 |
| 20 | Women | -0.95 | 0.933 | 1 | 180 | 1561 | 218 |
| 21 | Psychological Health | -0.05 | 0.6286 | 5 | 174 | 1524 | 217 |
| 22 | Intervention | -0.275 | 0.2107 | 4 | 180 | 1623 | 214 |
| 23 | Childhood Obesity | -0.5 | 0.3793 | 3 | 152 | 1376 | 204 |
| 24 | Adults | -0.275 | 0.1143 | 4 | 171 | 1284 | 181 |
| 25 | Body Image | -0.5 | 0.2915 | 3 | 162 | 1330 | 179 |
| 26 | Eating Disorder | -0.725 | 0.3324 | 2 | 163 | 1261 | 177 |
| 27 | Disorders | -0.05 | 0.1786 | 5 | 164 | 1110 | 150 |
| 28 | Validation | -0.725 | 0.8971 | 2 | 161 | 999 | 142 |
| 29 | Diet | -0.95 | 0.3387 | 1 | 167 | 907 | 137 |
| 30 | Self-Esteem | -0.5 | 0.7963 | 3 | 142 | 1048 | 136 |
